# Recent Developments in Polyurea Research for Enhanced Impact Penetration Resistance and Blast Mitigation

**DOI:** 10.3390/polym16030440

**Published:** 2024-02-05

**Authors:** Yifan Wang, Lailong Ding, Jiayu Lin, Xishun Qiu, Chao Wu, Changhao Liu, Yicheng Tian, Rui Zhang, Weibo Huang, Mingliang Ma

**Affiliations:** School of Civil Engineering, Qingdao University of Technology, Qingdao 266520, China; 18764811011@163.com (Y.W.); dinglailong1014@126.com (L.D.); 15064540744@163.com (J.L.); 15288903218@163.com (X.Q.); 15069829127@163.com (C.W.); changhao0538@126.com (C.L.); tianyicheng01@126.com (Y.T.); zhangray126@126.com (R.Z.); spua@163.com (W.H.)

**Keywords:** polyurea, blast-resistant, impact-resistant, ballistic penetration

## Abstract

Polyurea has gained significant attention in recent years as a functional polymer material, specifically regarding blast and impact protection. The molecular structure of polyurea is characterized by the rapid reaction between isocyanate and the terminal amine component, and forms an elastomeric copolymer that enhances substrate protection against blast impact and fragmentation penetration. At the nanoscale, a phase-separated microstructure emerges, with dispersed hard segment microregions within a continuous matrix of soft segments. This unique microstructure contributes to the remarkable mechanical properties of polyurea. To maximize these properties, it is crucial to analyze the molecular structure and explore methods like formulation optimization and the incorporation of reinforcing materials or fibers. Current research efforts in polyurea applications for protective purposes primarily concentrate on construction, infrastructure, military, transportation and industrial products and facilities. Future research directions should encompass deliberate formulation design and modification, systematic exploration of factors influencing protective performance across various applications and the integration of numerical simulations and experiments to reveal the protective mechanisms of polyurea. This paper provides an extensive literature review that specifically examines the utilization of polyurea for blast and impact protection. It encompasses discussions on material optimization, protective mechanisms and its applications in blast and impact protection.

## 1. Introduction

The escalating risk of terrorist attacks, military conflicts, explosive accidents and chemical disasters has heightened the necessity for blast and impact resistance in military and civilian buildings. This requirement is particularly crucial for military protective equipment such as military ships, armoured vehicles and protective helmets, all of which need to withstand shock waves and high-speed fragments resulting from near-field explosions [1,2,3,4,5,6,7,8,9,10]. Even though research on the protective properties of metal structures and high-performance fiber composites has made significant progress, there is an increasing need for lightweight and efficient blast protection structures [11,12,13,14,15,16,17,18]. To accommodate this requirement, the application of high-performance polymers in composite protection structures has gained momentum due to their enhanced protection performance, cost reduction and expanded application possibilities. In essence, the development and design of efficient protective materials and structures play a vital role in mitigating blast and impact threats, wherein the utilization of high-performance polymers in composite protection structures holds promising potential in meeting these challenges.

Polyurea is a block copolymer synthesized by rapidly reacting an isocyanate prepolymer with a polyamine. Commercial polyurea formulations typically comprise two components: Component A, an isocyanate prepolymer; and Component B, a blend of end-amino polyethers, chain extenders and various additives [19]. The presence of soft and hard segments in polyurea gives rise to a unique microphase separation phenomenon within its microstructure. This microstructure reveals that the hard segments are uniformly dispersed within the soft segment matrix, creating a cross-linked grid structure. As a result, polyurea can be regarded as a nanocomposite material, with the hard segments serving as reinforcements within the soft segment matrix. This distinctive microstructure imparts favorable macroscopic properties to polyurea, including stability, high strength and resistance to aging.

Originally developed and researched by Texaco (now Huntsman) in the mid-1980s, polyurea proves to be a highly efficient and cost-effective alternative to polyurethanes, as it possesses a diverse range of desirable properties. These include high strength, high toughness, rapid construction and minimal environmental impact, making polyurea a highly promising material for various applications. Notably, polyurea elastomer technology distinguishes itself from traditional coating methods due to its rapid curing speed, ease of application and ability to form thick coatings. Furthermore, polyurea materials provide exceptional protection and effectively mitigate the harm caused by explosive fragments due to their high toughness. Additionally, polyurea coatings are cost-effective and convenient to handle, rendering them a unique and advantageous choice for enhancing blast resistance in structures.

Currently, research and development efforts are concentrated on creating materials with both high strength and elongation. Multiple studies have shown that polyurea demonstrates a significant strain rate effect, signifying that its mechanical properties undergo substantial changes when subjected to high strain rate loads [19,20]. Indeed, the mechanical properties of polyurea exhibit distinct behavior under blast and shock loading compared to quasi-static conditions. This distinction is a crucial aspect of polyurea’s performance in blast and shock protection. While quasi-static mechanical properties can serve as a reference parameter, they are not the sole determinant. In practical applications, it is vital to consider the performance of polyurea in blast and shock scenarios to effectively address real-world requirements. By evaluating and understanding polyurea’s response under dynamic conditions, it becomes possible to optimize its design and formulation for enhanced blast and shock protection. This approach ensures that the material meets the specific demands and challenges posed by dynamic loading situations.

The present paper provides a comprehensive review on the research and application of polyurea in blast impact protection. It examines the optimization of materials specifically tailored for blast impact protection and the underlying mechanisms of protection and the diverse array of applications of polyurea in fields such as construction, infrastructure, military, transportation and industrial products and facilities. Furthermore, it explores the current research direction and development trends of polyurea in the realm of blast impact protection.

## 2. Structural Characteristics of Polyurea Molecules and Material Optimization

Polyurea demonstrates remarkable chemical stability and outstanding physical properties, rendering it highly suitable for blast and impact protection in diverse environments. To accommodate various substrates or structures, the ratio of hard and soft segments in polyurea can be adjusted by modifying the type and content of isocyanate and amino compounds [21,22,23,24,25,26]. Moreover, the incorporation of nano or micron particles and reinforcing fibers serves to enhance the mechanical properties and blast resistance of polyurea composites [11,12,13,14,15,16,17,18,27,28,29]. Additionally, molecular dynamics (MD) simulation can be utilized to optimize polyurea at the molecular level, further enhancing its performance. This comprehensive approach ensures the effectiveness and versatility of polyurea in applications related to blast and impact protection, exhibiting the desired levels of perplexity and burstiness in the content.

### 2.1. Structural Features of Polyurea Molecules

**Figure 1:**
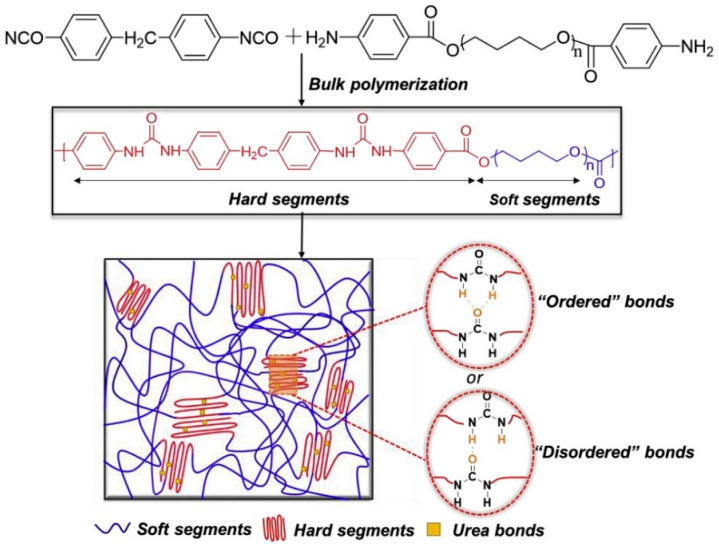
Synthesis and schematic representation of PUR1000 [32]. Reproduced with permission from [Ting Li, et al.], [A multi-scale investigation on effects of hydrogen bonding on microstructure and macro−properties in a polyurea.]; published by [Polymer], [2018].

Polyurea is a micro-phase-separated block polymer material composed of hard and soft segments. The hard segment consists of strongly polar urea-containing (-NH-CO-NH-) chain segments connected by hydrogen bonding and π-stacked aromatic chain segments. It is generated by the reaction of polyisocyanate and the chain extender, resulting in a glass transition temperature (Tg) above ambient temperature. The soft segment is composed of oligomeric polyol and oligomeric polyamines, providing flexibility and aliphatic chain segments. The Tg of the soft segment is typically below −30 °C. This structure makes polyurea a micro-dispersed thermoplastic cross-linked polymer at room temperature (20 °C). The soft segments exhibit superelasticity, while the hard segments display elastoplastic behavior. Hydrogen bonds are formed between the hard segments and between the hard and soft segments, creating reversible physical cross-linking and reinforcing components. These hydrogen bonds also contribute to the formation of a mesh structure [30], resulting in excellent mechanical properties such as modulus, hardness and tear strength. The soft segments contribute to the material’s flexibility and low-temperature resistance. MD simulations have shown that the soft segments can store more strain energy compared to the hard segments, while energy is consumed through structural disruption and hydrogen bond dissociation of the hard segments [31]. Polyurea (PUR1000), as synthesized by Ting Li et al. [32], was prepared through the native polymerization of polycarbodiimide-modified diphenylmethane diisocyanate and poly(tetramethylene oxide di-p-aminobenzoate). The synthesis pathways of the diisocyanate and diamine are depicted in Figure 1. In this process, P1000, comprising poly(tetramethylene oxide) (PTMO 1000) repeating units, serves as a soft chain segment in the polyurea, while the terminal phenyl ring and urea bond of the diamine are utilized as hard chain segments.

The length of the soft segments in polyurea has a significant influence on its mechanical properties both under quasi-static and dynamic conditions. When the length of the soft segments increases, the tensile strength decreases proportionally and the Tg decreases as well. In a study by D.A. Tzelepis et al. [33], several polyureas were synthesized with the same molecular weight of the soft segments but varying weight fractions of the hard segments. The structure of the polyurethanes was characterized using differential scanning calorimetry (DSC) and a transmission electron microscope (TEM) which revealed that the three polymers had almost the same Tg. According to the time-temperature superposition (TTS), a reduction in Tg makes the materials less susceptible to brittle damage by maintaining their elasticity during high strain rate loads, such as blast and impact loads.

The presence of urea bonds within the hard segments of polyurea plays a critical role in determining its properties. The size, properties and distribution of the hard segment region in the block copolymer can be manipulated to enhance the material’s loss spectrum. Researchers have conducted several studies focusing on the thermal stability of polyurea and have observed that it undergoes one-step decomposition at high temperatures. The thermal decomposition process of polyurea initiates with the breakdown of the urea bonds within the hard segments. The thermal decomposition temperature typically falls within the range of 300 to 320 °C. To improve the thermal stability of polyurea, di- and tri-functional polyamines can be incorporated into the formulation. These additives reinforce the cross-linked structure of the material, consequently enhancing its thermal stability. By strengthening the material’s structure, the added polyamines contribute to its ability to withstand higher temperatures without undergoing significant decomposition.

**Figure 2:**
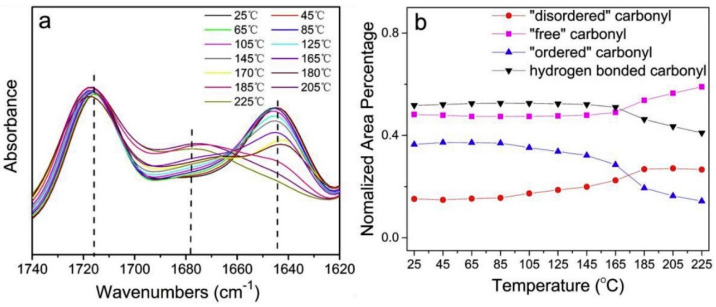
(**a**) Temperature−dependent FTIR spectra and (**b**) the corresponding percentage of different carbonyl groups of PUR1000 from 25 °C to 225 °C [32]. Reproduced with permission from [Ting Li, et al.], [A multi-scale investigation on effects of hydrogen bonding on microstructure and macro−properties in a polyurea.]; published by [Polymer], [2018].

Hydrogen bonds significantly influence the molecular structure and mechanical properties of polyureas. Unlike the mono-coordinated hydrogen bonding in urethane urethanes, polyurea exhibit bi-coordinated hydrogen bonding within the hard-segmented urea bonds, resulting in higher bonding energy and enhanced micro-phase segregation within the material. The redshift in the amino (N-H) and carbonyl (C=O) regions of the polymer provides valuable insights into the extent of hydrogen bonding. Techniques such as Fourier transform infrared spectroscopy (FTIR) can be used to analyze changes in the position and intensity of these regions, enabling researchers to estimate the strength and prevalence of hydrogen bonding in polyureas. Understanding the role of hydrogen bonding in polyureas is crucial as it significantly impacts various material properties. Bi-coordinated hydrogen bonding fosters micro-phase segregation, which influences the overall mechanical behavior and structural characteristics of polyureas, thus allowing for tailored properties to suit specific applications. Considering the complexities of hydrogen bonding and its effects on polyureas, researchers can further explore and optimize the material’s mechanical properties, thermal stability and chemical resistance. This knowledge creates opportunities for advancements and applications in a wide range of industries. The FTIR spectra in Figure 2 demonstrates the change in C=O group intensity as the temperature increases from 25 °C to 225 °C. As the temperature increases, an intriguing phenomenon occurs in polyurea. The intensity of the “ordered” C=O groups undergoes a decrease, accompanied by a shift in the peak towards a higher frequency (from 1645 cm^−1^ to 1655 cm^−1^). These changes indicate the dissociation and weakening of hydrogen bonds within the material. This behavior can be attributed to the dissociation of the ordered carbonyl groups present in the polyurea structure. The results obtained from the study suggest that with increasing temperature, there is a rearrangement of chain segments within the hard domain. Consequently, the ordered carbonyl groups decompose into disordered carbonyl groups [32]. The degree of hydrogen bonding in the polymer directly influences the energy storage modulus, which is a crucial factor closely associated with the mechanical properties of polyureas. Typically, a higher energy storage modulus correlates with better mechanical properties, however, when the hydrogen bonds in the hard segments of the polyurea are disrupted or broken, a significant decline in the material’s mechanical properties is observed. This information highlights the intricate relationship between temperature, hydrogen bonding and the mechanical behavior of polyurea. Understanding these dynamics is vital for optimizing the design and performance of polyurea-based materials in various applications.

**Figure 3:**
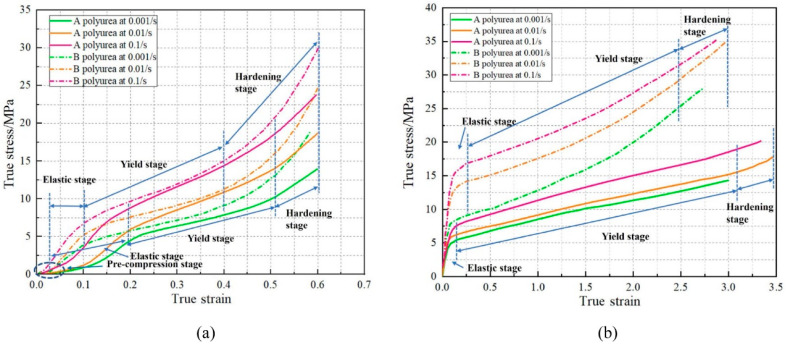
Results of (**a**) quasi-static compression tests; and (**b**) quasi-static tensile tests [34]. Reproduced with permission from [Zhang, L., et al.], [Effect of polyurea coating with different mechanical properties on blast resistance of aluminum alloy circular tube structures: Experiments vs numerical simulations.]; published by [Thin-Walled Struct.], [2023].

**Figure 4:**
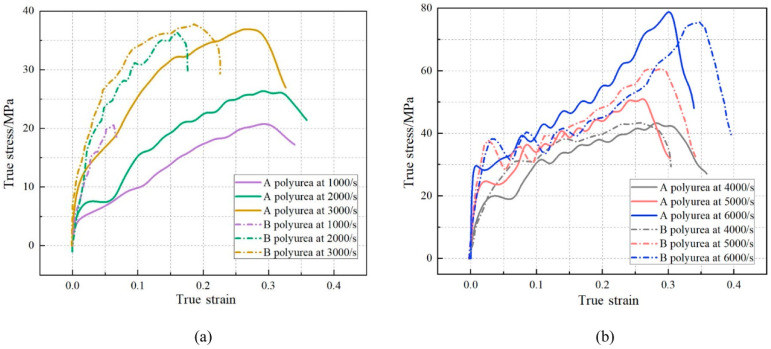
Split Hopkinson pressure bar (SHPB) results of polyurea at different strain rates, (**a**) strain rates in the range of 1000/s−3000/s, (**b**) strain rates in the range of 4000/s−6000/s [34]. Reproduced with permission from [Zhang, L., et al.], [Effect of polyurea coating with different mechanical properties on blast resistance of aluminum alloy circular tube structures: Experiments vs numerical simulations.]; published by [Thin−Walled Struct.], [2023].

Upon analyzing the microstructure, an interesting observation emerges. The cross-linked mesh structure of polyurea showcases a uniform dispersion of its hard segments within the matrix of soft segments. It is worth emphasizing that the formation of distinct phases is not solely dictated by the Tg, but is also influenced by the molecular weight of the soft segments. When the molecular weight of the soft segments surpasses a specific threshold, discrete hard segments start to manifest. This intricate internal microstructure of polyurea contributes significantly to its exceptional mechanical properties, which become evident on a macroscopic scale. When exposed to static or quasi-static tensile or compressive forces, polyurea exhibits fascinating superelastic behavior. It displays a remarkable tensile strength of up to 28 MPa, along with an elongation at break that can reach an impressive 1000%. Additionally, polyurea demonstrates tear strength ranging from 44 to 105 kN·m^−1^. This was also confirmed by L. Zhang et al. [34], who conducted tensile and compressive tests on two polyureas at various strain rates, as illustrated in Figure 3. These mechanical characteristics highlight the versatility and robustness of polyurea as a material. Its unique microstructure, with uniformly dispersed hard segments and soft segment matrix, enables it to withstand and absorb significant forces. This understanding of polyurea’s microstructure and mechanical behavior is pivotal for leveraging its potential in a wide array of applications including structural materials, coatings and protective layers. Under dynamic loading conditions, the mechanical behavior of polyurea becomes highly intricate, exhibiting several key characteristics that contribute to its enhanced explosion-proof impact resistance. These characteristics include nonlinearity in the stress-strain curve, high sensitivity to strain rate and temperature effects [35] and a strong dependence on pressure, as illustrated in Figure 4. In a study conducted by Guo et al. [36], a novel type of polyurea coating was synthesized using aromatic diphenylmethane diisocyanate, high molecular weight-terminated aminopolyethers and the appropriate additives. The mechanical properties of these polyurea coatings were evaluated using an electronic universal testing machine and Hopkinson’s compression rod technique. The results demonstrated a remarkable transition in the mechanical behavior of the polyurea coatings from a rubber-like state under quasi-static loading to a glass-like state under dynamic loading, effectively showing excellent impact resistance. The study further revealed that multiple factors including the ratio of soft to hard segment content, loading rate and temperature significantly influence the mechanical properties of polyurea. As the proportion of hard segments increases, polyurea undergoes a transition from a soft rubber state to a hard plastic state. Initially, the overall mechanical properties of polyurea improve with an increase in the hard segment content. However, there comes a point where further increases in the hard segment content result in a decline in the material’s mechanical properties. In addition, investigations into elastomer infiltration in polyurea-aluminum composite structures have highlighted the strong influence of test temperature on the mechanical response of polyurea elastomer under high-speed impacts. At higher temperatures, polyurea exhibits a rubbery state, while at lower temperatures, it transforms into a glassy state, showcasing distinct mechanical behaviors depending on the temperature conditions. These findings emphasize the intricate relationship between loading conditions, temperature and the mechanical response of polyurea. Understanding these complexities is crucial for designing and optimizing polyurea-based materials with tailored mechanical properties for various applications.

### 2.2. Optimization of Polyurea Composition

Polyurea, a highly versatile polymer, comprises both hard and soft segments in its composition. The hard segment is typically composed of isocyanates, while the soft segment consists of amino compounds. The properties of polyurea can be precisely controlled by manipulating the ratio of hard and soft. This can be achieved through varying the amount and type of isocyanates and amines. Increasing the concentration of isocyanates in polyurea leads to a higher proportion of hard segments, resulting in elevated hardness, strength and durability. Conversely, augmenting the content of amino compounds increases the ratio of soft segments, leading to enhanced flexibility, bendability and elasticity of polyurea. Thus, adjusting the isocyanate-to-amino compound ratio directly influences the balance between hard and soft segments within the polyurea structure. For example, elevating the proportion of isocyanates while reducing the amount of amino compounds can enhance the hardness and strength of polyurea, although at the expense of flexibility and bendability. This ability to tailor the ratio of hard and soft segments empowers the customization of polyurea properties to align with specific application requirements.

Different types of isocyanates and amino compounds have varying properties and reactivity. By selecting different combinations of these compounds, the ratio of hard and soft segments in polyurea can be adjusted, along with other properties such as heat resistance and chemical resistance. In a study by M.F. Sonnenschein et al. [21], polyether polyols were used as raw materials and an ester exchange reaction with p-amino benzoate was conducted to synthesize terminated aniline polyols. The resulting amine exhibited higher thermo-oxidative stability and viscosity compared to the parent polyol. The physical properties of polyurea/polyurethane elastomers prepared from these aniline-terminated end groups were evaluated in terms of reaction kinetics, tensile properties, morphology and aging properties. The study found that increasing the volume of hard segments in order to enhance elastomer hardness and tensile strength led to challenges in phase separation due to the inhomogeneous reactivity between the aniline end groups and the hydroxyl groups of the hard-segment chain extender. This hindered the desired phase separation of the hard segments.

The mechanical and thermal properties of PU and PUR coatings are significantly influenced by the length of the aliphatic chain and the properties of the aromatic chain extender [22]. In the study conducted by V. Shahi et al. [23], polyurethane elastomers were synthesized using PTMO-based diamines and MDI diisocyanate as raw materials via step-growth polymerization. The investigation of thermo-mechanical properties revealed that PU-HB05, with increased incorporation of long-chained diamines, exhibited lower thermal conductivity and heat capacity, a more amorphous structure and increased stability at high temperatures. In another study, H. Guo et al. [24] synthesized various polyurea coating materials by adjusting the proportions of amino-terminated polyether types and amine chain extender types in the original polyurea components. Analysis of the properties of these coatings led to the conclusion that the optimal ratio of amino-terminated polyether D2000 to T5000 was 12:1 and the optimal ratio of amine chain extender E100 to W6200 was 1.6:1, as these ratios resulted in the best performance indicators for the polyurea coating.

Covalent thermosets are known for their strong mechanical properties, however, they lack reprocessing or recycling capabilities, making them fragile. In a study by B. Qin et al. [25], a new approach was developed to enhance the toughness and recyclability of cross-linked supramolecular polyurethanes (CSPUs). This was achieved by introducing noncovalent bonds into the polymer backbone. CSPUs were prepared through the copolymerization of diisocyanate monomers, tetrahydrogen bonded diamine monomers and covalent diamine/triamine monomers. The resulting CSPUs exhibited excellent mechanical properties and solvent resistance due to the combination of covalent cross-linking and noncovalent bonding. Additionally, L. Zhang et al. [26] successfully synthesized a supramolecular polyurea elastomer by designing hydrogen bonding interactions with various strengths and incorporating permanent covalent bonds. This elastomer demonstrated remarkable mechanical strength with an elongation at break exceeding 1600%, a notch-insensitive tensile capacity of up to 800% and a toughness of up to 12,500 J m^−2^. The covalent cross-linking provided high strength, while the multi-strength hydrogen bonding offered elasticity, energy dissipation and fast self-healing properties at room temperature.

A crucial aspect to consider is that the modification of polyurea through the alteration of isocyanate and amino compounds requires meticulous handling. The reactivity and properties of these compounds can significantly influence the structure and characteristics of the resulting polyurea. Consequently, it is imperative to conduct thorough experimentation and testing throughout the preparation and modification processes of polyurea. These measures are essential to ensure the attainment of the desired properties and stability of the material. Careful attention and precision are vital to optimize the outcome and guarantee the reliability of the modified polyurea.

### 2.3. Introduction of Enhanced Materials

Polyurea materials have the potential to be optimized by incorporating various forms of reinforcing materials [37]. By introducing fiber reinforcing materials, particle filling materials, foam reinforcing materials and nano reinforcing materials, the properties of polyurea such as strength, hardness, abrasion resistance, temperature resistance and chemical resistance can be enhanced.

Among the fiber reinforcing materials, glass fibers, carbon fibers and aramid fibers, among others, are known to significantly improve the strength, stiffness and durability of polyurea while also enhancing its temperature and chemical stability [11]. Typically, these fibers are integrated into polyurea in the form of yarn or cloth, creating a composite material. Additionally, polyurea-based hybrid composites can be synthesized [12]. Previous research studies have demonstrated that polyurea-coated fiber-reinforced composites can enhance the impact resistance of concrete slabs [13,14]. Furthermore, the combination of polyurea coatings with carbon fibers and basalt fiber-reinforced polymer reinforcement techniques has shown positive effects in enhancing the blast resistance of urban utility tunnels [15]. The use of glass-fiber reinforced polyurea materials has also been found to enhance the bullet intrusion resistance of steel plates [16]. In summary, the incorporation of various reinforcing materials into polyurea holds great potential for improving its properties and expanding its application range. In a study conducted by J. Lv et al. [17], a hierarchical interfacial phase with high interfacial shear strength and toughness was created in an aramid composite through in situ grafting and foaming of polyurea on the fiber surfaces, as well as epoxy infiltration into the pores of the aramid composite. This resulted in the construction of a “rigid-flexible” interlocking three-dimensional interfacial structure, further increasing the interfacial shear strength and toughness of aramid fiber composites. N.V. Vuong et al. [18] developed different types of composites consisting of corrugated glass fibers/vinyl ester and polyurea using a conceptual composite panel inspired by mollusk shell pearl laminates. Various interlocking corrugated laminates were simulated and compared with planar and conventional dog-bone interlocking laminates, demonstrating a significant improvement in the performance of this composite under blast and impact loading.

Particulate fillers, including silica sand, alumina, carbon black and nanoparticles, are recognized for enhancing the hardness, abrasion resistance and durability of polyurea, as well as for improving its thermal and chemical stability [27]. A study conducted by A.S. Roy et al. [27] used a detailed all-atom MD model confirmed these results. Typically, granular filler materials are mixed into polyurea in the form of powder or granules. For instance, Q. Liu et al. [28] performed quasi-static and dynamic compression tests on pure polyurea and polyurea/SiC nanocomposites with varying amounts of nanofillers at different strain rates using an electronic universal testing machine and a SHPB device. The researchers found that, in comparison to pure polyurea, the addition of nanoparticles influences on the compression properties. Under static loading, the nanocomposites with the content 1.5 wt% fillers greatly affected the compressive mechanical properties. However, under dynamic loading, the mechanical behaviors of nanocomposites with the additional amount of SiC (0.7 wt%) was observed to be more active compared to other nanocomposites. The reason may be that more cracks were formed on the inside of the specimens with the increased content of particles under a high stain rate which leads to the decrease of mechanical properties.

Nano-reinforcement materials such as nano-oxides, carbon nanotubes and nanofibers, can enhance the strength, toughness and durability of polyurea while improving its thermal and chemical stability. These nano-reinforcement materials are typically incorporated into polyurea as nanoparticles. G. Wu et al. [29] developed a novel highly elastic protective coating by reinforcing polyurea with nano-silica filler composites. The polyurea material exhibited a tensile strength of 15.7 MPa and an elongation at break of 472%. Application of the polyurea coating resulted in a 9.7 kJ/m^2^ increase in the impact strength of the substrate, while maintaining good mechanical properties and ductility. Simulation results indicated that the polyurea coating could effectively mitigate the impact caused by the ball’s equivalent force at different velocities.

## 3. Protection Mechanism under Blast Impact Loading and Ballistic Penetration

Polyurea is a high-performance polymer with outstanding protective properties, making it suitable for resisting blast impact loading and ballistic penetration. Its protection mechanism encompasses several aspects. Firstly, the complex structure formed by the hard and soft segments of polyurea provides it with high strength and toughness. Consequently, when subjected to impact loading or ballistic penetration, polyurea effectively withstands external forces using its strength and toughness. Secondly, polyurea exhibits excellent energy absorption capabilities, allowing it to absorb and disperse the energy from external impact loading and ballistic penetration, thereby safeguarding the protected objects. Moreover, polyurea possesses the ability to undergo deformation in response to external forces, thereby dispersing and mitigating their effects and ultimately protecting the objects within. Additionally, polyurea’s chemical stability ensures that its performance remains unaffected under diverse environmental conditions. This stability prevents any chemical reactions or decomposition from occurring when exposed to external impact loading and ballistic intrusion, further contributing to the protection of the object.

### 3.1. Hydrogen Bond Dissociation and Reorganization, Rearrangement and Hardening of Soft and Hard Segments

Polyurea is known for its high strength, stiffness, hardness, flexibility and toughness, which can be attributed to the presence of hydrogen bonding within its molecules. The hardening of polyurea is achieved through the dissociation of hydrogen bonds and the reorganization of soft and hard segments via a heat curing reaction. During this reaction, the amide and urea bonds within the polyurea molecules are broken and reorganized, resulting in the formation of new hydrogen bonds and molecular chain cross-links. This cross-linking process enhances the strength and hardness of polyurea [31,38,39]. To investigate the temperature-dependent microscale impact response of polyurea at a fixed impact velocity, Y. Sun et al. [40] observed an increased absorption of localized impact energy at approximately 115 °C, which corresponds to the transition temperature from the glassy to the rubbery state when subjected to high-speed dynamic loading. Notably, materials that exhibit a wider temperature range in the glass transition zone and lower microphase segregation demonstrate superior flexibility and energy absorption under high strain rate loading conditions [41,42].

The soft phase exhibits superelasticity, while the hard phase demonstrates elastoplastic behavior. Through a combination of experimental and simulation analysis, M.H. Jandaghian et al. [43] discovered that the performance of the formulation in response to low-intensity impacts (such as seismic waves) is primarily influenced by the soft phase. On the other hand, the interaction between the two phases determines the formulation’s overall resistance against projectile penetration into the structure, with the hard phase playing a key role in response to high-intensity indirect impacts (such as blast shockwaves). The ductility of the material increases proportionally with the length of the soft section, while the tensile strength decreases as the length of the soft section increases. The frequency required to initiate the dynamic transition process from the “rubber” to “glass” state is directly proportional to the length of the soft section [44]. Remarkably, all analyzed formulations exhibit an elastic response even under typical high-frequency blast loading conditions.

Both the length of the soft segments and the type of hydrogen bonding significantly influence the impact response [45]. The impact-induced changes in the chain segments are primarily caused by bending and torsional bonding and the molecular potential energy is predominantly stored in the soft mid-segments. Upon impact, the ordered arrangement of the hard segments is disrupted, resulting in a reduction in the number of hydrogen bonds. The dissociation of hydrogen bonds leads to a substantial increment in the potential [41,46]. Additionally, the soft phase stores a greater amount of strain energy compared to the hard phase under impact. Conversely, the hard phase dissipates plastic energy through hydrogen bond dissociation and structural disruption, which is more prominent at stronger shocks [47]. Polyureas can undergo hardening through the dissociation of hydrogen bonding and rearrangement of the soft and hard segments via a light-curing reaction. In this reaction, the amide and urea bonds within the polyurea molecule are fragmented and restructured to create new hydrogen bonds and molecular chain cross-links. As a result, the polyurea molecules become interconnected, enhancing the strength and hardness of the material. This reaction necessitates a specific light intensity and duration, typically achieved through ultraviolet or visible light irradiation.

### 3.2. Viscous Dissipation and Strain Rate Effects within the Material

The viscous dissipation and strain rate effects within polyurea materials primarily depend on the structure and movement mode of the polyurea molecules themselves. The polyurea molecule consists of two distinct structural units: the hard segment and the soft segment. The hard segment is formed through the reaction of diisocyanate and diol, resulting in a polyurethane structural unit with high strength and stiffness. In contrast, the soft segment is formed through the reaction of long-chain diol and dibasic acid, giving rise to a polyester structural unit with high flexibility and toughness. This combination of hard and soft segments imparts polyurea with both strength and flexibility, enabling it to undergo deformation when subjected to external stresses and thereby consuming energy [48]. The dissipation of shock wave energy occurs through three mechanisms: (1) thermal dissipation, (2) viscous dissipation and (3) plastic dissipation. Heat is dissipated due to viscosity and internal friction within the material. Viscous dissipation refers to the incomplete relaxation of molecular chains in a short period of time, resulting in the retention of potential energy. Plastic dissipation primarily occurs in the hard phase of the material. The mesoscale inhomogeneous two-phase structure must undergo deformation coordination during loading, leading to a significant lateral displacement of the soft phase. This displacement increases the deformation energy and frictional heat of the molecular chains [47]. Yao et al. [46] discovered that polyurea with a lower content of hard segments exhibits higher energy dissipation when the shock is released under the same impact pressure. The main mode of energy dissipation is through heat dissipation, which arises from an increase in kinetic energy. Unlike in a tensile simulation, under impact loading the increase in molecular potential energy is primarily partitioned into the increments of bonding energy, angular energy and dihedral angular energy, with the majority of these increments stored in the soft segments. During high-velocity impacts, the increment in hydrogen bonding potential accounts for only around 1% of the internal energy increment.

The motion mode of polyurea molecules plays a significant role in their viscous dissipation and strain rate effects. The presence of hydrogen bonding between polyurea molecules causes the bonds to break and rearrange under external stress, resulting in various modes of motion such as rotation, slippage and twisting. These modes of motion generate friction and sticking between the polyurea molecules, leading to viscous dissipation and strain rate effects. Notably, the strain rate effect becomes more pronounced at higher strain rates [20,49,50,51]. An increase in strain rate leads to higher rheological stress, compressive strength, strain rate sensitivity and strain energy, which can enhance the protection of structures against blast and shock loading [28,52,53]. Wu et al. [54] conducted an investigation on the enhancement properties of coated polyurea on localized damage of 6063-T5 aluminum alloy tubing using static and dynamic mechanical property tests, explosion tests and numerical simulation calculations. Their findings reveal that the AP103 polyurea exhibits a strain-rate sensitive effect during tensile testing, with a noticeable elastic phase followed by a slight strain-hardening phase. In dynamic compression experiments, the polyurea exhibits a significant nonlinear stress-strain relationship. At low strain rates, polyurea displays superelastic properties, whereas at high strain rates, it exhibits clear yield slip, strain-hardening properties and strain rate effects.

In conclusion, the viscous dissipation and strain rate effects in polyurea are predominantly influenced by the molecular structure and motion modes. These effects contribute to polyurea’s remarkable energy absorption and stability capabilities under high stress-strain rates, making it highly promising for a wide range of applications requiring high strength and high speed.

### 3.3. Impedance Mismatch between Base Material and Polyurea

Impedance mismatch occur when there are interfacial reflections and transmissions between the substrate and polyurea leading to potential problems like energy loss and signal attenuation. This mismatch is primarily caused by variations in physical parameters such as dielectric constant, acoustic wave velocity and density between the substrate and polyurea. Insufficient interfacial adhesion is a common source of impedance mismatch, resulting from factors such as differences in chemical composition, surface morphology and roughness of the substrate surface. These factors can weaken the bond between the materials and give rise to issues like interfacial peeling, crack expansion and material separation [55,56,57]. Another cause of impedance mismatch is the disparity in coefficient of thermal expansion between the substrate and polyurea. When temperature changes occur, the substrate and polyurea may undergo different degrees of thermal expansion, resulting in stress and strain discrepancies. This mismatch can lead to interfacial shear stresses, stress concentrations and subsequent material damage and degradation. Differences in mechanical properties between the substrate and polyurea also contribute to impedance mismatches. For example, the substrate may possess higher stiffness and strength, while the polyurea exhibits greater toughness and energy absorption. This mismatch can result in interfacial stress concentrations and failures, impacting the overall performance of the material [58,59,60,61]. Insufficient chemical compatibility between the substrate and polyurea can give rise to impedance mismatch as well. This chemical mismatch may cause issues such as interfacial reactions, dissolution or corrosion, ultimately affecting the performance and durability of the material. Surface energy differences between the substrate and polyurea can also contribute to impedance mismatches. These disparities can make processes such as coating, bonding or wetting more challenging, thereby influencing the interfacial properties and durability of the material.

From a macroscopic standpoint, the impedance mismatch between the polyurea and the substrate is the primary factor contributing to the polyurea’s resistance to explosion and impact. To mitigate the degradation of material properties resulting from this mismatch, enhancing the interfacial strength between the polyurea and the substrate can be pursued [56]. Additionally, when it is not feasible to alter interfacial properties due to disparities in materials and construction methods, a quantitative design of impedance mismatch can be employed to attain the desired protective objectives [62]. The deliberate creation of impedance mismatch aims to strike a balance between material properties and energy absorption. In the design of protective multilayer armour, T. Rahimzadeh et al. [62] utilized finite element analysis and determined that the outer layer of the armour should have a higher acoustic impedance than its neighboring layers. This allows for multiple reflections at the interface between the two layers, effectively tuning the wave. However, it is essential to ensure that the impedance mismatch is not excessive, as this can result in inefficient transmission of the stress wave across successive layers.

**Figure 5:**
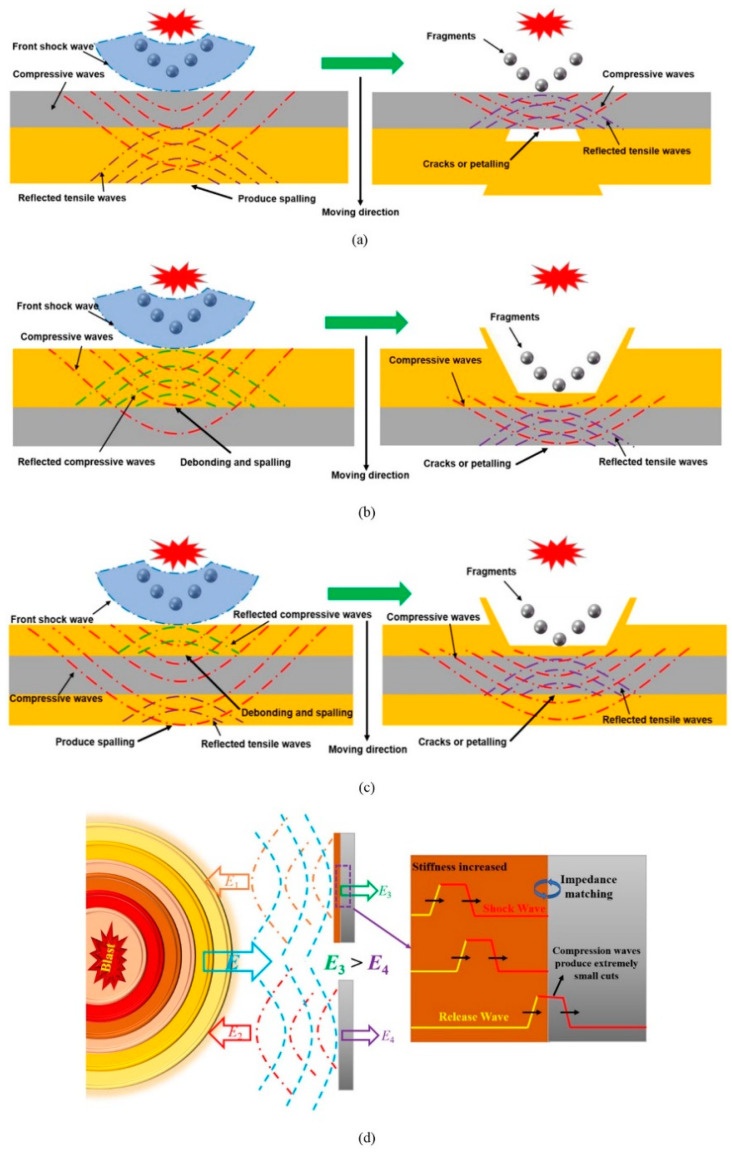
Stress wave propagation in PCS plates. (**a**) Rear−side sprayed, (**b**) impact−side sprayed and (**c**) both−side sprayed plates with shock wave propagation effect, (**d**) reasons for the enhanced steel plate damage effect of PCS plates with thinner polyurea coatings on the impact side [64]. Reproduced with permission from [Wu, G., et al.], [Damage response of polyurea−coated steel plates under combined blast and fragments loading.]; published by [J. Constr. Steel. Res.], [2022].

Low-thickness polyurea coatings have been found to increase the frequency of wave reflections at the high-impedance polyurea/steel interface, leading to a significant increase in the pressure level and instantaneous specific energy density of the polyurea [63]. However, increasing the elastic resistance of the steel plate has the opposite effect on blast resistance. In a field explosion test conducted by G. Wu et al. [64], it was observed that when the impact side was sprayed with a thin polyurea layer, the unloading wave inside the polyurea layer could not catch up with the loading wave in time. Consequently, the compression wave carrying more energy passed through the polyurea layer and directly impacted the steel plate, resulting in more severe damage. Figure 5 depicts the propagation of the stress wave in the PCS plate. The bonding strength between the polyurea layer and the steel plate also plays a crucial role in impact resistance. Premature debonding of the polymer from the substrate can prevent the coating from maximizing its energy absorption effect [65]. L. Zhang et al. [66] investigated the blast resistance of ASTM 1045 steel plates reinforced with polyurea of varying mechanical properties and observed that the early overall collapse of high ductility polyurea coated on the front side of the plate severely limited the protective effect of the polyurea. Consequently, the damage to the plate was not significantly reduced under loading. The high ductility polyurea coating on the front side optimized the impedance relationship within the target, reducing the reflected load and attenuating the damage through the unloading effect of the loaded wave. However, the effectiveness was diminished due to the debonding of the polyurea and steel plate. Coating the backside of the target plate with highly ductile polyurea allowed for the dissipation of impact energy within an appropriate timeframe while reflecting and unloading the stress wave. This greatly improved the explosive resistance of the target plate.

To investigate the interfacial impedance at the nanoscale. Y. Chen et al. [67] conducted MD simulations to analyze the process of excitation wave premelting and dispersion of single-crystal copper when subjected to cylindrical convergent impacts. Their findings revealed that the premelted zone near the free surface experienced spalling off after unloading due to the formation of a stretching zone near the free surface, caused by the interaction between the reflected wave and the unloaded wave. The impact damage can be effectively mitigated by the presence of a polymer layer on the impact surface at the nanoscale. However, the reverberations of the shock wave weaken the polymer layer on the back side. In a separate study, M.A.N. Dewapriya et al. [68] performed MD simulations of ballistic impact tests on multilayered nanostructures. The results showed that the ultrathin polyurea layer applied to the impact surface effectively redistributes the impact load to the underlying metal layer, resulting in improved energy absorption.

## 4. Research and Application of Polyurea in Blast/Impact Protection

Polyurea, as a novel protective material, has been extensively studied in various fields due to its exceptional protective properties. For instance, polyurea blast-resistant sheets are employed to safeguard buildings, industrial plants and mining facilities during explosive events. Also, polyurea impact-resistant materials are used to protect individuals and equipment from impacts and collisions. Polyurea bulletproof vest materials are effective in preventing bullet or shrapnel penetration in order to keep people safe. Polyurea blast buffer materials are utilized to attenuate blast shockwaves on buildings and equipment. Additionally, polyurea sheets provide explosion and shock protection for buildings and equipment, effectively averting damage and destruction caused by explosions or shock waves. Polyurea can be categorized based on its applications in various sectors such as buildings and infrastructure, transportation, military and industrial facilities. Furthermore, based on the type of protective substrate, polyurea can be classified into single-layer substrate protection and protection of laminated composite materials and structures.

### 4.1. Construction and Infrastructure

The explosion and impact resistance of civil engineering structures under extreme conditions, such as explosions, earthquakes, storms and more, plays a vital role in ensuring their safe and stable operation. High-performance polymer materials with exceptional strength, toughness and durability are well-suited for this purpose. The fracture mechanism of these materials, when subjected to contact explosions, encompasses various factors, including the high-temperature mechanical property destruction mechanism, high-temperature impact load coupling fracture mechanism, high-speed load brittle fracture mechanism and tensile fracture mechanism [41,42]. These mechanisms collectively contribute to the material’s behavior under extreme conditions.

**Figure 6:**
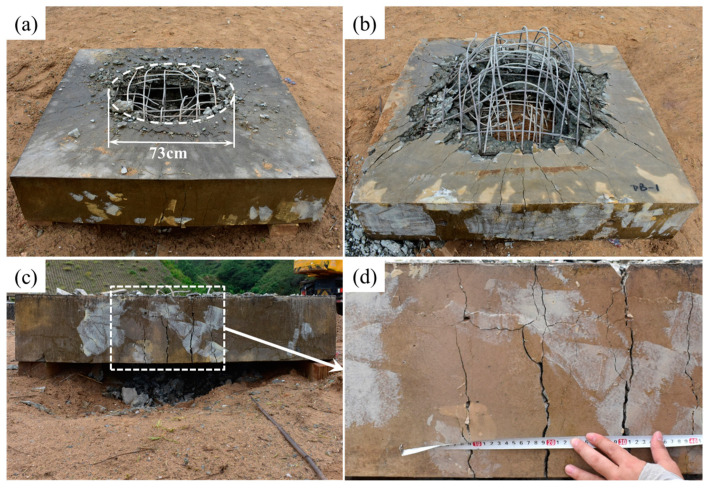
Uncoated concrete specimen after explosion test deformation diagram: (**a**) blast face; (**b**) back−blast face; (**c**) side face; and (**d**) crack area of the side face [41]. Reproduced with permission from [Huang, W., et al.], [Study of Blast Mitigation Performance and Fracture Mechanism of Polyurea under Contact Explosion.]; published by [Polymers], [2022].

**Figure 7:**
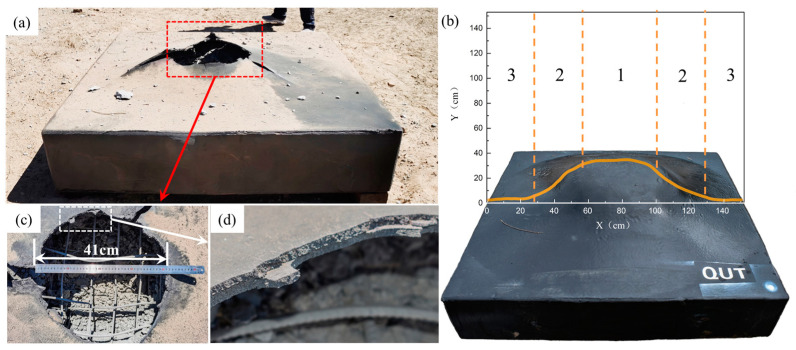
T26 polyurea−coated concrete specimen after explosion test deformation diagram: (**a**) blast face; (**b**) back−blast face and cross-sectional view of back−blast face, and the backside was divided into three areas 1, 2 and 3 according to the deformation; (**c**) damage area of the blast−face coating; and (**d**) detail of coating damage [41]. Reproduced with permission from [Huang, W., et al.], [Study of Blast Mitigation Performance and Fracture Mechanism of Polyurea under Contact Explosion.]; published by [Polymers], [2022].

Polyurea is gaining significant attention in the construction industry as a structural retrofit and reinforcement material due to its ability to be easily sprayed onto the surfaces of structural members without the need for an epoxy bond interface [69]. Additionally, composite sandwich structures can be created to effectively handle damage across various situations [70,71]. Studies and simulations have demonstrated that plain reinforced concrete (NRC) protected with polyurea experiences reduced damage and residual displacement, showcasing its efficacy in enhancing blast resistance in NRC panels [41,42,72,73,74,75,76,77]. Autoclaved aerated concrete (AAC) panels, known for their lightweight nature, exceptional thermal insulation and energy absorption properties, are ideal materials for protecting structures. To enhance the blast resistance of AAC panels, Y.S. Chen et al. [78] implemented three strengthening schemes by applying 4 mm thick polyurea coatings to different areas of the reinforced AAC panels. Their findings indicated that the blast resistance of polyurea-coated AAC panels primarily relied on the bottom coating. With its excellent adhesion and ductility properties, polyurea coating proves to be an effective solution for reinforcing existing blast-resistant structures. W. Huang et al. [41] studied the protective performance of T26 polyurea on reinforced concrete (RC) slabs using a 10 kg trinitrotoluene (TNT) contact explosion test. The T26 polyurea coating was applied using PHX-40 proportioner (PMC Global, Inc., Branford, CT, USA), with the reaction volume ratio of component A to component B set at 1:1. The reaction temperature was maintained at 65 °C by preheating the equipment, and the spray pressure was controlled at 2500 psi. Subsequently, after allowing the T26 polyurea spray film specimens to cure in an environment with a temperature of 23 ± 2 °C and relative humidity of 60 ± 15% for seven days, the production of T26 polyurea protection for RC panels was completed. Upon comparing Figure 6 and Figure 7, it can be observed that the coated specimen exhibited a significantly reduced fragmentation rate compared to the unprotected specimen when subjected to a 10 kg TNT contact explosion, effectively achieving zero fragmentation on the anti-explosive surface of the protection.

**Figure 8:**
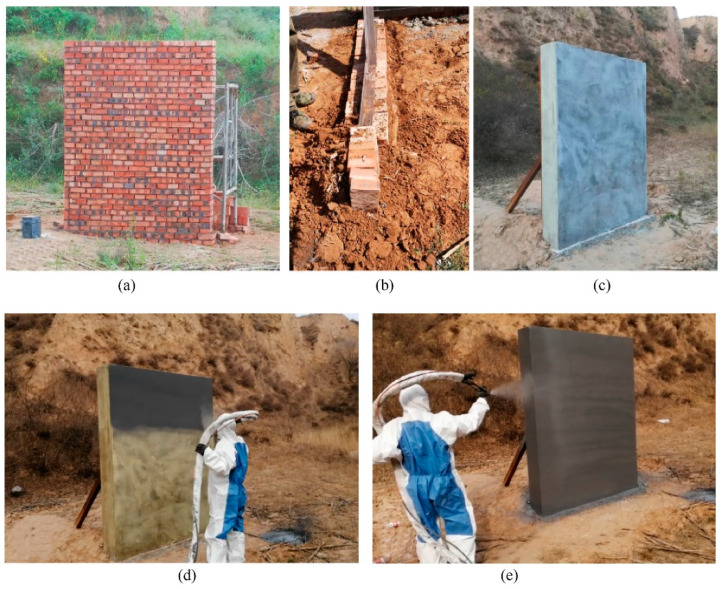
Construction of polyurea reinforced CBMU walls: (**a**) bare clay brick wall substrate; (**b**) foundation; (**c**) masonry wall with cement mortar; and (**d**,**e**) polyurea coating reinforcement [84]. Reproduced with permission from [Wu, G., et al.], [Blast response of clay brick masonry unit walls unreinforced and reinforced with polyurea elastomer.]; published by [Def. Technol.], [2022].

In the absence of reinforcement, concrete masonry wall structures are brittle with low flexural strength, making them unable to absorb strain energy. Consequently, the consequences of a terrorist attack on such structures can be extremely severe. Recognizing this issue, the U.S. Air Force Laboratory (AFRL) conducted tests from 1995 onwards to investigate the use of carbon fibers and aramid fibers for enhancing the blast resistance of concrete masonry walls. However, the high production costs and complex construction procedures associated with fiber composite materials made them impractical for large-scale use. As a result, AFRL began exploring the feasibility of using polymeric materials for masonry wall reinforcement from 1999 onwards. In this study, AFRL assessed 21 types of polymers, categorizing them based on the molding process into extrusion molding, spray molding and brush molding. The selection of the most suitable material for masonry wall repair was polyurea, which was evaluated comprehensively based on its chemical properties, mechanical properties, combustion properties and more. In 2001, AFRL conducted 12 explosion tests to assess the effectiveness of polyurea reinforcement on masonry walls. These experiments demonstrated that when a layer of polyurea was sprayed on the back-blast side of a masonry wall (with the direct shock wave acting on the face of the blast), it enhanced the blast resistance of the wall. This finding has been corroborated by multiple researchers [79,80,81,82]. L. Ji et al. [80] discovered that sprayed polyurea elastomers on a 240 mm wall resulted in improved blast resistance, as the polyurea layer enclosed the damaged areas and fragments within it. Increasing the thickness of the polyurea layer to 8 mm further enhanced the blast resistance, reducing the damaged area of the masonry wall by 55.6% compared to an unreinforced wall. Similarly, M. Gu et al. [81] found that double-sided spraying of polyurea and increasing its thickness on the back surface during a gas explosion improved detonation resistance. Z. Li et al. [83] examined the performance of carbon fiber-reinforced polymer (CFRP)-reinforced clay brick masonry walls under a gas explosion through field tests and numerical simulations. The results indicated that after reinforcement, the response and damage modes of the masonry walls changed from unidirectional to bidirectional. The reinforced carbon fiber fabric fractured at the edge and mid-span regions, with a centralized arrangement proving more effective in enhancing blast resistance compared to a decentralized arrangement. Furthermore, the application of sprayed polyurea improved the tensile effect, blast resistance and ultimate strain of the masonry wall. Several other researchers [84,85,86] have also verified the blast protection capabilities of polyurea elastomers for both reinforced and unreinforced clay brick masonry walls. Figure 8 illustrates the construction process of polyurea-reinforced CBMU walls.

Restraining concrete using fiber-reinforced polymer (FRP) composites has become a prominent method for reinforcing RC columns to enhance their axial properties. However, the full strain capacity of the reinforcing material may not be utilized due to stress concentrations resulting from concrete deformation or irregularities on the concrete surface. To address this issue, E. Akın et al. [87] enhanced the effectiveness of FRP restraint by applying a polyurea coating between the concrete and the FRP. The findings demonstrated that the polyurea coating led to a more uniform stress distribution within the material, thus increasing its ultimate strain capacity. Furthermore, the improvement of FRP restraint effectiveness through the utilization of polyurea was particularly evident in cyclic tests. J.-H. Song et al. [88] conducted a study that demonstrated the ability of fiber-reinforced polyurea (FRPU) composites to provide high load-bearing capacity to concrete girders. FRPU effectively prevents the deterioration of ductility in concrete girders and minimizes debonding of concrete surfaces. The reinforced specimens maintained a significant amount of load-carrying capacity and flexural ductility, even after the destruction of concrete on the tensile face in the mid-span section. Furthermore, the application of polyurea coatings positively influenced the cracking state of bent RC beams, although it had a minimal impact on their flexural strength [89].

**Figure 9:**
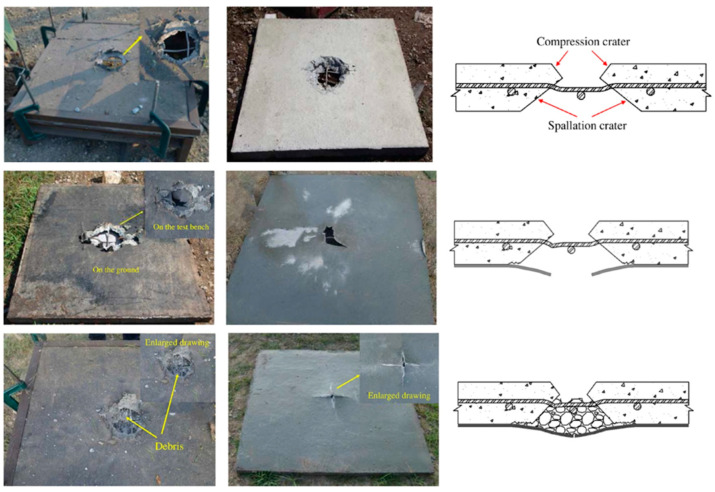
Plain RC plate specimen (**top**), PU−reinforced RC plate specimen (**middle**) and PU−WGF−reinforced RC plate specimen (**bottom**) after testing: before (**left**), after (**middle**) and cross−sectional view (**right**) [91]. Reproduced with permission from [Shi, S.Q., et al.], [Behavior of polyurea−woven glass fiber mesh composite reinforced RC slabs under contact explosion.]; published by [Int. J. Impact Eng.], [2019].

Glass fiber is commonly used as a reinforcement material for repairing and renovating concrete elements. Glass fiber reinforced polyurea (GFRPU) is a composite that combines polyurea with various fibers. Polyurea provides ductility, while fibers offer increased stiffness and strength. Test results have demonstrated the feasibility of using GFRPU as a reinforcement method to enhance load carrying capacity and flexural ductility [90]. GFRPU prevents sudden spalling and damage to concrete by debonding and it is applied through spraying, making it rather simple. GFRPU not only enhances load carrying capacity but also improves flexural ductility, however, the effectiveness of GFRPU in blast resistance is limited. Consequently, S.Q. Shi et al. [91] proposed a reinforcement method that involves adding woven glass fiber mesh (WGF) to a pure polyurea (PU) coating to form a PU-WGF composite coating. Through field blast tests conducted on plain RC panels, PU-reinforced RC panels, PU-WGF-reinforced RC panels and contact blast tests on PU-WCF composite carbon fiber mesh (PU-WCF)-reinforced RC panels, the study verified that the addition of WGF improves the force transmission, integrity and fracture resistance of PU coatings. Furthermore, it changes the damage mode of PU coatings from shear punching-shear damage to tensile damage, thereby greatly enhancing blast resistance performance. The results from testing plain RC plate specimens, PU-reinforced RC plate specimens and PU-WGF-reinforced RC plate specimens are presented in Figure 9.

The effectiveness of polyurea coatings and carbon fiber and basalt fiber reinforced polymer reinforcement techniques in enhancing the blast resistance of urban utility tunnels has been established in [15]. Y. Liu et al. [92] conducted blast tests to investigate the impact of polyurea coatings on the seismic performance of RC arch structures. The results demonstrated that polyurea coatings exhibit superior spalling resistance compared to the CFRP reinforcement method. This significantly improves the blast resistance of concrete arches, with closed polyurea coatings yielding the best results. The thickness and location of the polyurea reinforcement layer have a significant influence on the blast resistance of the structure. While polyurea reinforcement in the middle of the arch is a relatively effective method of blast resistance, solely increasing the thickness of polyurea does not guarantee an improvement [93]. Q. Liu et al. [94] examined the ballistic resistance of polyurea-coated CFRP panels subjected to projectile impact loading. The findings revealed that the polyurea coating on the back side of the CFRP plate substantially increased the ballistic resistance of the CFRP plate in terms of residual velocity, energy absorption ratio, deformation and damage level in comparison to the pure CFRP composite. Conversely, the polyurea coating caused more damage to the frontal side than the pure CFRP sheet.

### 4.2. Military and Defense

The military and defense sectors face numerous threats and combat environments including blast impact, shrapnel and bullet penetration. To enhance the protection of military equipment and facilities, researchers are exploring the development of innovative materials capable of addressing these challenges. Notably, there has been considerable research interest in blast and impact-resistant polyurea within the military and defense fields owing to its exceptional mechanical properties and energy absorption characteristics. Polyurea materials possess high strength, toughness and excellent energy absorption capabilities, enabling them to effectively absorb and disperse energy from blast impact and shrapnel penetration. As a result, they help minimize damage to military equipment, military vehicles and protective facilities.

Polyurea exhibits over 91% shock wave overpressure attenuation when subjected to explosions, making it suitable for various applications including explosion-proof protective panels [66,95,96,97,98]. D. Mohotti et al. [99] conducted an experiment where they exposed steel sheets coated with polyurea to near-field blast loading. The steel sheets, made of mild steel (XLERPLATE 350) and high-strength steel (BIS80), were coated with polyurea (6 mm and 12 mm thickness) on either the front or back side. The experiment was validated using three-dimensional scanning and numerical simulation. The results indicate that applying polyurea on the backside reduces residual deformation by around 20% and prevents the polyurea layer from melting. Conversely, the frontal coating can create additional spacing between the steel plate and the blast. L. Zhang et al. observed that adding a high hardness polyurea coating to the front surface of the steel plate improves impact resistance to some extent [66]. Applying a high hardness polyurea coating to the back side of the steel plate significantly enhances the steel plate’s explosion resistance, making it the most effective option. However, this coating may result in fragments flying backward, potentially causing secondary injuries. Applying high ductility polyurea coatings to both the front and back of the steel plate improves the steel plate’s explosion resistance, although the front coating is slightly less effective than the back coating. Generally, applying highly ductile polyurea coating to the back of the steel plate is the most preferable option.

Coating the impact surface of steel plates with polyurea material significantly enhances their elastic resistance in the context of ballistic materials [63,100]. According to an experimental study by P. Zhang et al. [101], coating mild alloy steel with polyurea enhances its ballistic resistance against cubic breakers. The study also found that the use of a high hardness polyurea coating on the front side of the target plate yields the best ballistic resistance. Y.-x. Sun et al. [16] observed that applying a polyurea coating to the surface of a steel plate enhances its resistance to penetration. Additionally, they noted that the velocity of the projectile decreases as the thickness of the polyurea coating increases. G. Toader et al. [102] developed polyurethane-polyurethane multi-walled carbon nanotubes (MWCNTs) nanocomposites with enhanced mechanical properties. The homogeneity of the nanocomposite film and the uniform dispersion of the nanofillers within the polymer matrix were verified through SEM and microCT studies. Improved thermal and mechanical properties were demonstrated through TGA, DSC, DMA and tensile tests. Impact tests on aluminum plates coated with urethane-polyurethane MWCNTs nanocomposites using a Hopkinson rod device confirmed the ability of the nanocomposite layer to maintain the integrity of the metal plate. P. Si et al. [1] assessed the ballistic performance of polyurea-reinforced ceramic/metal armour using the criterion of mass efficiency. The study found that the polyurea-coated armour exhibited 89% higher mass efficiency compared to the ceramic/metal armour, demonstrating superior ballistic performance and lighter weight. Utilizing polyurea interlayers is a prudent option for substantially enhancing the penetration resistance of sandwich panels [71,103]. Due to their exceptional impact resistance, polyurea foams have garnered significant attention from researchers involved in military applications, particularly body armour [104,105,106,107,108].

In practice, however, this is often accompanied by dual damage from blast and fragmentation penetration. Research has shown that the initial load, which causes significant deformation or damage to the structure, usually dominates [2]. When fragmentation penetration occurs first, it is crucial to have excellent ballistic properties per unit thickness at the front of the structure in order to enhance the combined ballistic and blast performance of the plate. Under the combined effect of blast and high-velocity fragmentation of polyurea-coated steel plates, the protective properties of the plates exhibit an inverse strengthening effect when the coating thickness is within a certain range, regardless of the configuration used. However, as the polyurea thickness increases, this reverse strengthening effect weakens. It is only when the polyurea thickness reaches a specific value that the strengthening effect occurs. Therefore, the thickness of the polyurea coating is crucial in determining its desired role. When the polyurea layer is too thin, the release wave is unable to catch up with the loading wave in the polyurea, resulting in the compression wave carrying more energy and directly acting on the steel plate, exacerbating the damage. In the case of target plates coated on one side, premature debonding and stress concentration of the steel plate also adversely affect its protective performance. However, when coated with polyurea on both sides, the debonding time is delayed which results in a relatively even stress distribution of the target plate and a more continuous and stable energy absorption process [3]. L. Zhang et al. [4] discovered that the improvement in the protective capability of polyurea coated on steel plates does not increase linearly with the thickness. Polyurea coatings with a thickness greater than 4 mm on one side enhance the protection against composite action. The thicker the coating, the higher the protective capacity. Coating the front and rear surfaces with 6 mm thick polyurea reduced maximum damage deflection by 20.8% and 18.5%, respectively. On the other hand, when the coating thickness is 2 mm, the degree of damage exacerbates, with the center area displaying petal damage. Coating both sides with polyurea does not reduce the damage degree of the target plate, regardless of the polyurea thickness. Microscopic study revealed that the front-coated polyurea underwent significant ablation from the blast products, greatly reducing its energy absorption efficiency. Under tensile stress waves, the polyurea coated on the back side experienced tensile fracture. The breaking of hydrogen bonds within the polyurea molecule is the key to the energy absorption of polyurea.

Blast traumatic brain injury (BTBI) is the most common injury among frontline combat soldiers [5]. The use of a helmet delays the impact of the shock wave on the wearer’s head, with the helmet absorbing some of the energy through the action of the aramid fiber visor and shell as well as the compression of the internal foam [6]. Kevlar is a high-performance synthetic fiber material widely used in ballistic protection due to its high strength and lighter weight compared to traditional materials like sheet metal or ceramics. Kevlar is more than 15 times stronger than steel and also has excellent energy absorption capabilities, effectively dispersing and mitigating the force of impacts to provide better protection. Additionally, its fiber structure withstands impact and disperses energy, reducing damage and penetration [7]. However, Kevlar helmets have limitations in blast resistance. The currently used advanced combat helmets (ACH) are primarily designed to maximize protection from ballistic impact and hard surface collisions. To enhance blast resistance, polyurea-based external coatings are used on the ACH [8,9]. Researchers like M. Grujicic et al. have quantified the ballistic performance of helmets using finite element analysis, aiming to design helmets with a better combination of ballistic and blast resistance. C.P. Chang et al. have developed a new high-strength composite laminate that is lighter and thinner than traditional Kevlar laminates while still meeting the NIJ Bulletproof Vest 010106 Level IIIA standard [9]. To address the issue of uneven mixing commonly encountered during the preparation of shear thickening fluids (STF), a planetary mixer was employed to blend the solid dispersed particles with the liquid dispersing medium. Subsequently, the shear thickening liquid was rolled using a three-roll mixer to ensure thorough dispersion of the solid particles within the shear thickening liquid. In the pursuit of further improvements, J. Lv et al. [17] have constructed a hierarchically structured interfacial phase in aramid composites by in situ grafting and foaming of polyurea on the fiber surface, as well as infiltrating epoxy resin into the pores. This process generates a large number of covalently bonded interfaces by filling the rigid resin matrix with soft, porous polyurea, forming a three-dimensional “rigid-soft” interlocking interface. The axial covalent bonding between the fiber and polyurea, along with the radial covalent bonding between polyurea and epoxy resin, increases the interfacial shear strength by 115.9%. Consequently, the newly formed interface exhibits higher energy consumption during interfacial destruction, leading to a significant increase in interfacial shear toughness (GIC) by 493.2% [17]. Furthermore, C.H. Shih et al. conducted ballistic tests to investigate the impact resistance of aramid fabrics reinforced with STFs, epoxy resins or polyurea elastomers. According to test results, the aramid composite structure treated with polyurea elastomer absorbed the most impact energy per unit area and demonstrated the best impact resistance [10].

STF have high energy absorption capabilities. However, relying solely on them to enhance protection from elastic cascades has limitations as it often increases the weight and thickness of the sample. Ballistic test results indicate that composites made from polyurea/Kevlar fabric exhibit superior impact resistance compared to conventional Kevlar fabrics. The development of high-strength composite laminates utilizing polyurea/Kevlar sheets combined with STF structures has resulted in samples that are over 17% thinner and lighter than conventional Kevlar laminates. In their study, Q. He et al. [109] achieved an impressive protective Kevlar/STF/STG composite with synergistic effects that make this lightweight, thickened and shear material-reinforced Kevlar fabric highly impact-resistant. These findings demonstrate significant potential for applications in portable protection. Similarly, A. Haris et al. [110] conducted research to explore the impact mitigation capability of a potential personal protective equipment (PPE) suspension mat made of polyurea and STF. By replacing a 20 mm thick conventional foam mat made from flexible polyurethane foam with a 4 mm thick polyurea mat for Twaron fabric samples ranging in thickness from 2 mm to 18 mm, the normalized peak pressure and impulse were reduced by approximately 74% and 49%, respectively. The study findings provide conclusive evidence that polyurea mats display the most effective shockwave mitigation performance. Therefore, polyurea holds potential for implementation as a suspension pad in PPE requiring shockwave mitigation capabilities such as fabric ballistic undershirts, bomb suits and combat helmets.

**Figure 10:**
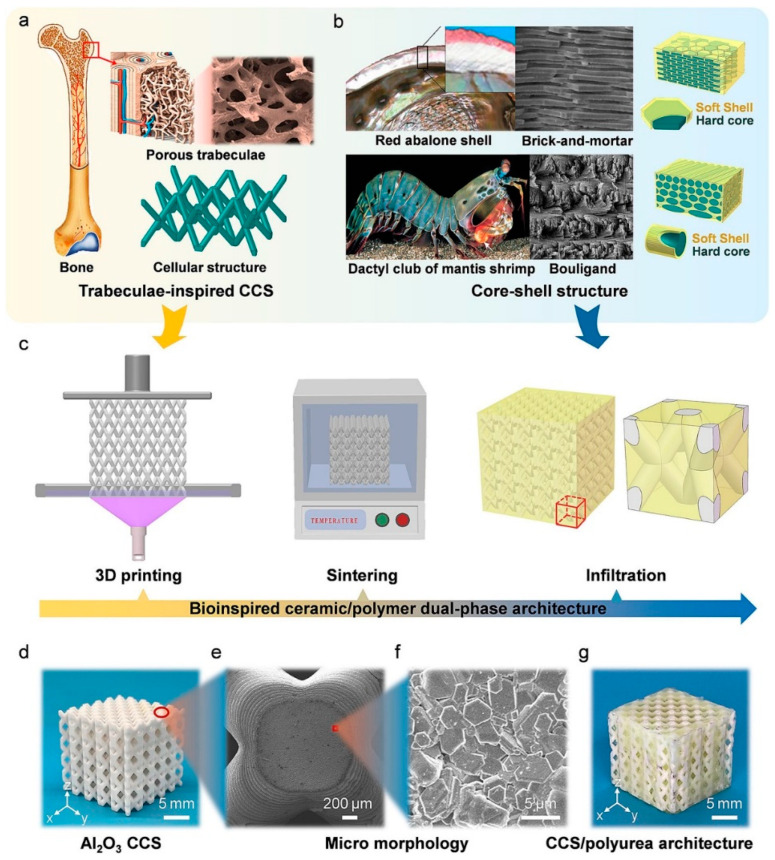
(**a**,**b**) Inspiration, (**c**) fabrication and (**d**–**g**) morphology of bioinspired CCS/polyurea dual-phase architecture [118]. Reproduced with permission from [Zhang, X., et al.], [3D-printed bioinspired Al2O3/polyurea dual-phase architecture with high robustness, energy absorption, and cyclic life.]; published by [Chem. Eng. J.], [2023].

The application of polyurea in the bionic field holds great potential. By combining the unique properties of polyurea materials with bionic principles, it is possible to develop new functional materials and devices that offer innovative solutions for bulletproofing challenges. S. Jafari et al. [111] developed an improved model for a ceramic-polyurea aluminum target based on the strain energy and fracture strain of the ceramic-polyurea layer. The results were verified using the improved Florence equation and numerical simulation was used to design the optimal thickness of each layer in the ceramic-polyurethane-aluminum composite armour [112]. The application of polyurea can effectively enhance the protective performance of ceramic/metal armour [113,114]. G. Zou et al. [115] designed a composite plate that consists of alumina ceramic/polyurea elastomer (PUE)/glass fiber reinforced plastic (GFRP) with a surface density of 3.6 g/cm^2^. It was observed that the ceramic layer plays a crucial role in energy dissipation during bullet penetration, while the PUE layer influences penetration time. The penetration time of the PUE layer was consistently longer than that of the ceramic layer, accounting for approximately 50% of the total penetration time. Furthermore, as bullet velocity increases, the energy absorption distance between PUE and ceramic gradually decreases. Y. Xiao et al. [116] prepared a pearl-layered “brick-mortar” structure of composite laminates by staggered layering of segmented ceramic/polyurea laminates with adhesive bonding of polyurea. These pearl-layered ceramic/polyurea composite panels with a “brick-mortar” structure combine the advantages of laminated and segmented composite panels, resulting in improved resistance to multiple impacts. Traditional honeycomb ceramic materials used in bulletproof materials show premature failure and poor impact resistance when subjected to high-speed projectiles. However, the use of square pore honeycomb ceramics filled with reinforcing materials in the honeycomb structure of ceramic matrix composite plates significantly enhances the toughness of the material. This composite material, compared to materials with the same thickness consisting of only honeycomb ceramic, improves energy-absorbing efficiency by 115.6% and energy-absorbing density by 52.3%, effectively addressing the brittleness issue of ceramic materials [117]. X. Zhang et al. [118] proposed a biomimetic cellular ceramic/polyurea (CCS/polyurea) biphasic structure using three-dimensional printing and infiltration techniques. This is depicted in Figure 10. This structure exhibits a specific load carrying capacity and energy absorption capacity that is 2.22 and 50.34 times higher than CCS, respectively, under quasi-static compressive loading. Multilayer polymer/ceramic nanocomposites offer excellent mechanical properties, heat resistance, corrosion resistance and protective characteristics, while being lightweight [119]. These properties make composites a crucial option for manufacturing advanced military equipment and improving soldier safety [120].

### 4.3. Transportation

Transportation systems play a crucial role in modern society and ensuring their safety and reliability is a challenge that cannot be overlooked. Traffic accidents and blast events can result in casualties, vehicle damage and infrastructure destruction, causing significant social and economic impacts. To enhance the safety and resilience of transportation systems against external shocks, researchers have turned their attention to developing innovative materials that can tackle these challenges. Blast and impact resistant polyurea has become a focal point of research due to its exceptional mechanical properties and energy absorption characteristics. With its high strength, toughness and abrasion resistance, polyurea materials are capable of effectively absorbing and dispersing impact energy, thereby mitigating damage to both individuals and facilities resulting from accidents and explosions [121].

The research and application of blast and impact-resistant polyurea in transportation systems holds immense significance. It offers potential solutions to enhance the impact resistance of transportation systems, mitigating the effects of accidents and explosive events on individuals and facilities. This focus on polyurea development and implementation contributes to overall safety and reliability. Moreover, it presents an opportunity to safeguard lives and property, foster sustainable transportation systems and prioritize the well-being of individuals. We can pave the way for resilient and secure transportation infrastructure by incorporating blast and impact-resistant polyurea.

Explosion- and impact-resistant polyurea is widely utilized in safeguarding vehicle structures and equipment including body impact beams, crash cushions and ballistic materials. The exceptional impact resistance of polyurea materials effectively absorbs and disperses collision energy, reducing injuries and damage in accidents. In the marine environment, severe conditions and corrosiveness challenge the durability of military equipment for sea missions. To enhance the survivability of naval structures against underwater explosions, L.H. Dai et al. conducted a study [122] on blast resistance using thin steel sheets coated with polyurea. The study investigated factors influencing the explosion resistance performance of the metal substrate by testing different coating positions (front and back) and thicknesses. The polyurea coating minimized deformation in the blast test compared to the bare steel plate. The thickness of the coating, material properties and bond strength between the substrate and coating can impact the protective effect. Notably, the polyurea coating exhibited consistent protection even under strong fluid impact [123].

Explosion and impact-resistant polyurea has widespread applications in the aerospace industry. It is utilized for fabricating resilient materials for aircrafts and spacecrafts, protecting them from external impacts and blast events during takeoff, landing and flight. The lightweight properties of polyurea materials enhance aircraft performance while ensuring reliable blast and impact protection. The application of polyurea coatings on the foam aluminum underside of the aircraft enhances ballistic resistance, absorbing and dispersing energy and alleviating deformation and damage caused by explosions or impacts [124]. Additionally, coating impact surfaces with polyurea effectively enhances the impact resistance of polyurea-coated aluminum alloy (PCAA) panels [125,126]. This coating provides an extra layer of protection which improves the overall durability and toughness of the panel, therefore enabling it to withstand substantial impacts and reduce potential damage.

**Figure 11:**
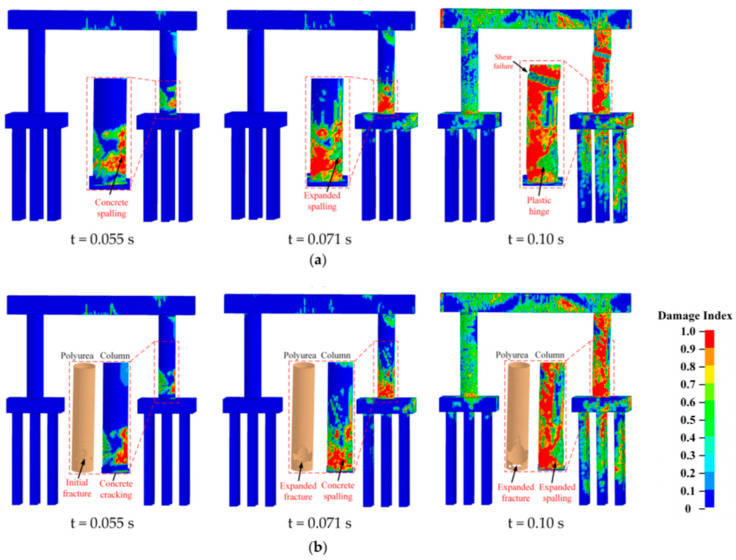
Damage statuses and propagations: (**a**) bare pier and (**b**) polyurea−retrofitted pier [128]. Reproduced with permission from [Chen, F., et al.], [Multi−Hazard−Resistant Behavior of CFRP− and Polyurea−Retrofitted Reinforced Concrete Two−Column Piers under Combined Collision−Blast Loading.]; published by [Materials], [2023].

Bridge columns play a crucial role in bridge structures and are constantly exposed to mechanical and chemical influences from various loads and environmental factors. However, the long-term use and exposure to external environmental effects can result in surface damage, corrosion and deterioration of bridge columns, consequently diminishing their structural performance and service life. To combat these issues, the application of polyurea coatings to protect and repair bridge column surfaces has been identified as an effective solution. This approach improves the durability, corrosion resistance and structural safety of bridge structures, while also extending their service life and providing reliable and safe transportation infrastructure [55]. The effectiveness of in situ retrofit solutions for RC bridge columns using FRP cladding or polyurea coatings was investigated by C. Fang et al. [127] under combined collision-bursting loading conditions. The findings of the study demonstrated that the composite coatings of FRP and polyurea were successful in mitigating the effects of vehicle collision and air blast. C. Fang and colleagues [128] validated the protective performance of polyurea against combined collision-explosion loading on RC bridge piers through numerical simulation, as depicted in Figure 11.

### 4.4. Industrial Products and Facilities

The industrial products and facilities sector is susceptible to a range of risks such as external shocks, vibrations and explosions during their operation and use, resulting in equipment damage, production interruptions and potential injuries. Industries such as mineral development are prone to fire hazards, which have led to an increased demand for materials with antistatic properties [129]. Nonetheless, enhancing antistatic properties may compromise other performance aspects, thereby restricting their application.

Steel storage tanks are commonly used in various industries for storing chemicals, water, oil and petroleum products. These tanks can be prone to internal explosions caused by flammable vapor clouds as well as external explosives [130]. Ensuring the explosion-proof performance of steel tanks is crucial when storing volatile liquids. In an effort to investigate methods for improving the explosion resistance of steel tanks, a study by Y.X. Jiang et al. [131] examined the response of both monolithic steel tanks and tanks coated with polyurea under the influence of explosive loads through on-site blasting tests using TNT explosives. The findings indicated that the polyurea coating effectively reduced the maximum and residual displacements of the tanks. Additionally, Y.X. Jiang et al. [132] conducted gas explosion experiments and utilized numerical and theoretical models to investigate the response and energy absorption mechanism of steel tanks while also analyzing the impact of polyurea coating. The results demonstrated that the blast load energy was primarily absorbed through plastic hinge line bending and tension in the connection zone. The polyurea coating primarily influenced the tank displacement by increasing the area density and bending moment of the tank.

Tanks play a critical role as fuel components in various mechanical equipment, however, they are highly vulnerable to dynamic loads such as explosions and debris. The impact of blast fragments on liquid-filled containers generates hydrodynamic impacts that can cause localized damage and overall deformation of the container. To address this issue, polyurea coatings have emerged as a rapid and effective means of enhancing the protection of liquid-filled containers, including fuel tanks [133]. In a study by G. Wu et al. [134], two types of polyurea materials—AMMT-53 and AMMT-55—were designed and experimentally investigated for their performance in protecting tanks with varying thicknesses of polyurea coatings under the combined effects of blast shockwaves and debris. The findings revealed that these two polyurea materials exhibited distinct protective properties. While AMMT-53 effectively reduced the perforation rate, it did not prevent liquid leakage. AMMT-55 did not significantly reduce the perforation rate, but its self-healing property proved effective in preventing liquid leakage. Numerical simulation results corroborated these findings, further supporting the effectiveness of the coated polyurea layer in protecting fuel tanks. X. Wang et al. [135] also demonstrated, through experiments and numerical simulations, that polyurea not only reduced the perforation rate and damage area on the tank but also inhibited the leakage of internal fluids due to its excellent self-healing properties.

Aluminum plates, tubes and related products are extensively utilized in various industrial facilities, underscoring the critical importance of safeguarding polyurea coatings, particularly in situations involving potential hazards such as accidental explosions. In their study, Mostofi and colleagues [136] conducted experimental and numerical analyses to assess the influence of a polyurea coating as a reinforcing layer on the dynamic plastic response and resistance of aluminum plates under gas burst loading. The experiments entailed subjecting rectangular aluminum plates to gas detonation forming (GDF) using a single-stage device. The researchers compared the residual deformation of polyurea-coated aluminum (PU-Al) plates with that of single-layer aluminum plates of the same areal density. The research findings indicated that the total pre-blast pressure had the most significant effect on the central permanent deflection at 39.4%. Furthermore, the thickness of the polyurea coating and the aluminum plate influenced the central permanent deflection by 29.7% and 20.3%, respectively. The study demonstrated that coating application notably decreased the central permanent deflection of the metal plates, with the PU-Al structure outperforming the uncoated aluminum plate, particularly at higher total pre-blast pressures. Additionally, a separate study by another researcher [34] revealed that polyurea significantly improves the explosion resistance of aluminum alloy round tubes when proper bonding is achieved.

Port terminals, cross-sea tunnels and offshore working platforms play essential roles as economic trade and logistics hubs, but are also susceptible to underwater threats such as diving explosives and mines. Therefore, the development and implementation of underwater explosion protection technology is of paramount importance. Considering its exceptional mechanical properties, protective characteristics and resistance to corrosion, polyurea demonstrates significant potential for use in safeguarding port terminals against underwater explosions. Polyurea coatings can be employed to provide an additional protective layer on various surfaces within port terminal facilities including floors, walls and pipelines [57,137,138,139]. Additionally, polyurea coatings possess exceptional impact resistance, corrosion resistance and flame-retardant properties that enable them to efficiently absorb and dissipate energy from blasts, effectively mitigating damages and injuries caused by explosions. E. Gauch et al. [140] conducted experiments and computational simulations to examine the response of composite cylinders to underwater near-field explosion (UNDEX) loading, specifically focusing on the influence of polyurea coatings. The results indicated that the internal energy and overall strain of the material exhibited an increase proportional to the coating thickness, thereby demonstrating the cylinder’s reduced damage through the application of polyurea coatings. J. Liu et al. [141] observed that thin steel plates subjected to underwater explosions experienced deformation or even cracking, while the application of polyurea coating on the surface effectively delayed metal necking failure and enhanced explosion resistance. The protective effectiveness of polyurea is influenced by factors such as strength, thickness and spraying method. Optimally, a three-fold coating thickness compared to the steel plate is advised when applying polyurea on the front surface.

AISI 304 steel finds extensive applications in various industries such as the food industry, home appliances, consumer electronics, medical devices, construction, decoration, hardware tools, automotive sector, petrochemical industry, water treatment plants and aerospace. Furthermore, the application of polyurea coating can significantly enhance the impact resistance, adhesion and fatigue life of AISI 304 steel [142]. Previous research [143] has indicated that applying a polyurea coating to both the top and bottom surfaces of a steel plate enhances its impact resistance. The polyurea layer on the lower side of the composite plate enhanced its impact resistance through the neck hysteresis effect. Additionally, the polyurea layer on the upper side of the composite plate reduces stress concentration, thereby delaying or preventing fracture.

## 5. Conclusions and Outlook

Polyurea, as a novel functional material, has seen significant advancements in blast and intrusion resistance. The material has garnered substantial interest in military, security and protection domains due to its exceptional impact resistance and protective properties. In recent years, polyurea’s properties have surpassed conventional coatings in areas such as tensile/compression strength, elongation at break, tear strength, strain rate sensitivity and energy absorption, which can enhance the stability of various structures and materials under dynamic loading. This positions polyurea as a potential alternative to current materials for reinforcing existing structures.

Despite the noticeable improvement in polyurea’s properties due to the design of new materials and the use of new raw materials, there are drawbacks to its application. The protective effect of polyurea is typically thickness-dependent, Thinner coatings may offer inadequate protection and excessively thick coatings can add unwarranted weight and complexity to the structure. Additionally, polyurea is susceptible to aging, deterioration or damage from environmental and service conditions such as prolonged exposure to ultraviolet light, high temperatures, humidity and chemicals. Moreover, the construction of polyurea necessitates specific technical requirements and specialized knowledge to prevent issues like unevenness, cracking and inadequate adhesion, which could compromise its protective performance. Furthermore, the cost of spray-coated polyurea elastomers is relatively high, encompassing material, construction and maintenance expenses. While these drawbacks impact the application of polyurea in practical protection projects, the material’s advantages in blast and impact protection compared to polyurethane, epoxy resin and other available materials are considerable. With reasonable modification and structural design, its shortcomings can be addressed to enrich its functionalization.

Currently, extensive research on protective properties of polyurea concentrates on modifying existing structures to meet the requirements of specific applications. Moving forward, integrating polyurea as an essential protective material with structural design is a trend in joint material and structural development. Polyurea can be further developed to have multifunctional properties by introducing functional fillers or surface coatings to realize multifunctional protective effects for diverse fields. The engineering application of polyurea is also a future development direction, with further optimization of its spraying process and coating properties to enable large-area, high-efficiency applications. These areas of exploration will undoubtedly attract the attention of researchers and academics in the forthcoming years.

## Data Availability

Not applicable.

## Dual-use Research Statement

We have made the following statements to our paper titled “Recent Developments in Polyurea Research for Enhanced Impact Penetration Resistance and Blast Mitigation”.

Ø Explanation of Potential Risks: Our paper examines the protective properties of armor materials. The research is limited to providing some theoretical and experimental support for the development of impact dynamics only and does not pose a threat to public health or national security.
Ø Evaluation of Benefits to the General Public: Our research is limited to the academic field, which is beneficial to the development of material science. There is no risk to the general public.
Ø Compliance with Laws: As an ethical responsibility, we strictly adhere to relevant national and international laws about dual-use research. And we have considered and adhered to these regulations in our paper.

## References

[1] Si P., Liu Y., Yan J., Bai F., Huang F. (2022). Ballistic Performance of Polyurea-Reinforced Ceramic/Metal Armor Subjected to Projectile Impact. Materials.

[2] Chu D., Wang Y., Yang S., Li Z., Zhuang Z., Liu Z. (2023). Analysis and design for the comprehensive ballistic and blast resistance of polyurea-coated steel plate. Def. Technol..

[3] Zhang L., Ji C., Wang X., Wang Y., Wu G., Zhu H., Han Z. (2022). Strengthening and converse strengthening effects of polyurea layer on polyurea–steel composite structure subjected to combined actions of blast and fragments. Thin-Walled Struct..

[4] Zhang L., Wang X., Wang Y., Gu J., Ji C., Wu G., Cheng L. (2022). High-hardness polyurea coated steel plates subjected to combined loadings of shock wave and fragments. Lat. Am. J. Solids Struct..

[5] Tse K.M., Bin Tan L., Bin Sapingi M.A., Franklyn M., Lee P.V.S., Tan V.B.C., Lee H.P. (2017). The role of a composite polycarbonate-aerogel face shield in protecting the human brain from blast-induced injury: A fluid–structure interaction (FSI) study. J. Sandw. Struct. Mater..

[6] Valverde-Marcos B., Rubio I., Antona-Makoshi J., Chawla A., Loya J.A., Rodríguez-Millán M. (2020). Numerical Analysis of EOD Helmet under Blast Load Events Using Human Head Model. Appl. Sci..

[7] Grujicic M., Snipes J.S., Ramaswami S. (2018). Multi-scale computational analysis of the nano-indentation and nano-scratch testing of Kevlar (R) 49 single fibers. Proc. Inst. Mech. Eng. Part L-J. Mater.-Design Appl..

[8] Grujicic M., Ramaswami S., Snipes J., Dudt P. (2016). Potential improvement in helmet blast-protection via the use of a polyurea external coating: Combined experimental/computational analyses. Proc. Inst. Mech. Eng. Part L J. Mater. Des. Appl..

[9] Chang C.-P., Shih C.-H., You J.-L., Youh M.-J., Liu Y.-M., Ger M.-D. (2021). Preparation and Ballistic Performance of a Multi-Layer Armor System Composed of Kevlar/Polyurea Composites and Shear Thickening Fluid (STF)-Filled Paper Honeycomb Panels. Polymers.

[10] Shih C.-H., You J.-L., Lee Y.-L., Cheng A.-Y., Chang C.-P., Liu Y.-M., Ger M.-D. (2022). Design and Ballistic Performance of Hybrid Plates Manufactured from Aramid Composites for Developing Multilayered Armor Systems. Polymers.

[11] Chundawat T., Vaya D., Sini N., Varma I. (2016). Blast mitigation using FRP retrofitting and coating techniques. Polym. Compos..

[12] Petre R., Zecheru T., Petrea N., Ginghina R., Sandu S., Muresan M., Matache L.C., Sava A.C., Neatu F. (2018). Synthesis and Mechanical Properties of Polyurea-Based Hybrid Composites for Ballistic Individual Protection. Mater. Plast..

[13] Li H., Wang D., Xiao Z., Qin Z., Xiong J., Han Q., Wang X. (2022). Investigation of vibro-impact resistance of fiber reinforced composite plates with polyurea coating with elastic constraints. Aerosp. Sci. Technol..

[14] Li H., Wang D., Zhang H., Wang X., Qin Z., Guan Z. (2022). Optimal design of vibro-impact resistant fiber reinforced composite plates with polyurea coating. Compos. Struct..

[15] Zhou J.-N., Chen X.-S., Zhou Y.-Z., Wang W.-Y., Wang P., Kong X.-L., Xu Y., Geng H.-S., Jin F.-N. (2022). Blast responses of polyurea retrofitted utility tunnel reinforced with basalt fibre reinforced polymer bars. Def. Technol..

[16] Sun Y.-X., Wang X., Ji C., Zhao C.-X., Liu P.-L., Meng L., Zhang K., Jiang T. (2021). Experimental investigation on anti-penetration performance of polyurea-coated ASTM1045 steel plate subjected to projectile impact. Def. Technol..

[17] Lv J., Liu Y., Qin Y., Yin Q., Chen S., Cheng Z., Yin J., Dai Y., Liu Y., Liu X. (2021). Constructing “Rigid-and-Soft” interlocking stereoscopic interphase structure of aramid fiber composites with high interfacial shear strength and toughness. Compos. Part A Appl. Sci. Manuf..

[18] Nguyen-Van V., Wickramasinghe S., Ghazlan A., Nguyen-Xuan H., Tran P. (2020). Uniaxial and biaxial bioinspired interlocking composite panels subjected to dynamic loadings. Thin-Walled Struct..

[19] Zhang R., Huang W., Lyu P., Yan S., Wang X., Ju J. (2022). Polyurea for Blast and Impact Protection: A Review. Polymers.

[20] Cui J., Shi Y., Zhang X., Huang W., Ma M. (2021). Experimental study on the tension and puncture behavior of spray polyurea at high strain rates. Polym. Test..

[21] Sonnenschein M.F., Virgili J.M., Larive M.Z., Wendt B.L. (2018). Synthesis of aniline-terminated polyethers and resulting polyurethane/polyurea elastomers. J. Polym. Sci. Part A Polym. Chem..

[22] Toader G., Moldovan A.E., Diacon A., Dirloman F.M., Rusen E., Podaru A., Rotariu T., Ginghina R.E., Hoza O.E. (2023). Effect of Aromatic Chain Extenders on Polyurea and Polyurethane Coatings Designed for Defense Applications. Polymers.

[23] Shahi V., Alizadeh V., Amirkhizi A.V. (2020). Thermo-mechanical characterization of polyurea variants. Mech. Time-Dependent Mater..

[24] Guo H., Du C., Chen Y., Li D., Hu W., Lv X. (2022). Study on protective performance of impact-resistant polyurea and its coated concrete under impact loading. Constr. Build. Mater..

[25] Qin B., Zhang S., Sun P., Tang B., Yin Z., Cao X., Chen Q., Xu J., Zhang X. (2020). Tough and Multi-Recyclable Cross-Linked Supramolecular Polyureas via Incorporating Noncovalent Bonds into Main-Chains. Adv. Mater..

[26] Zhang L., Wang D., Xu L., Zhang X., Zhang A., Xu Y. (2020). A highly stretchable, transparent, notch-insensitive self-healing elastomer for coating. J. Mater. Chem. C.

[27] Roy A.S., Mitra N., Ghosh S. (2022). Investigating the molecular origins of deformation in polyurea. Polymer.

[28] Liu Q., Chen P.-W., Guo Y.-S., Su J.-J., Han L., Arab A., Yuan J.-F. (2021). Mechanical behavior and failure mechanism of polyurea nanocomposites under quasi-static and dynamic compressive loading. Def. Technol..

[29] Wu G., Fang Z., Qin X., Fu J. (2022). Preparation and Properties of Impact Resistant Polyurea Coating for Fluorochemical Pipeline. Processes.

[30] Kumar N., Gupta P.K., Khilari S., Ranganath K.V.S. (2023). Synthesis, characterization and catalytic application of functionalized polyureas. J. Polym. Res..

[31] Rosenbloom S.I., Yang S.J., Tsakeredes N.J., Fors B.P., Silberstein M.N. (2021). Microstructural evolution of polyurea under hydrostatic pressure. Polymer.

[32] Li T., Zhang C., Xie Z., Xu J., Guo B.-H. (2018). A multi-scale investigation on effects of hydrogen bonding on micro-structure and macro-properties in a polyurea. Polymer.

[33] Tzelepis D.A., Suzuki J., Su Y.F., Wang Y., Lim Y.C., Zayernouri M., Ginzburg V.V. (2023). Effect of aromatic chain extenders on polyurea and polyurethane coatings designed for defense applications. J. Appl. Polym. Sci..

[34] Zhang L., Wang X., Ji C., Wang Y., Yang G., Zhao C., Tao C. (2023). Effect of polyurea coating with different mechanical properties on blast resistance of aluminum alloy circular tube structures: Experiments vs numerical simulations. Thin-Walled Struct..

[35] Akl W., Ali M., Aldraihem O., Baz A. (2021). Dynamic behavior of polyurea composites subjected to high strain rate loading. Finite Elem. Anal. Des..

[36] Guo H., Zhou Z., Gu M., Yu A., Ling X., Yao W. (2021). Preparation of impact-resistant functional polyurea coatings and effect of γ-ray irradiation on its microstructure and performance. Prog. Org. Coat..

[37] Goswami A., Das Adhikary S. (2019). Retrofitting materials for enhanced blast performance of Structures: Recent advancement and challenges ahead. Constr. Build. Mater..

[38] Manav M., Ortiz M. (2021). Molecular dynamics study of the shock response of polyurea. Polymer.

[39] Iqbal N., Tripathi M., Parthasarathy S., Kumar D., Roy P.K. (2016). Polyurea coatings for enhanced blast-mitigation: A review. RSC Adv..

[40] Sun Y., Kooi S.E., Nelson K.A., Hsieh A.J., Veysset D. (2020). Impact-induced glass-to-rubber transition of polyurea under high-velocity temperature-controlled microparticle impact. Appl. Phys. Lett..

[41] Huang W., Zhang R., Wang X., Lyu P., Ju J., Gao F., Yan S. (2022). Study of Blast Mitigation Performance and Fracture Mechanism of Polyurea under Contact Explosion. Polymers.

[42] Zhang R., Huang W., Lyu P., Sun P., Fang Z., Wang R. (2022). Study on the performance of blast-mitigation polyurea and fracture mechanism of the coated reinforced concrete slabs under contact explosion. Adv. Eng. Sci..

[43] Jandaghian M.H., Kazerooni H. (2020). Performance of polyurea formulations against impact loads: A molecular dynamics and mechanical simulation approach. J. Appl. Polym. Sci..

[44] Iqbal N., Tripathi M., Parthasarathy S., Kumar D., Roy P.K. (2018). Tuning the properties of segmented polyurea by regulating soft-segment length. J. Appl. Polym. Sci..

[45] Sun Y., Wu Y.-C.M., Veysset D., Kooi S.E., Hu W., Swager T.M., Nelson K.A., Hsieh A.J. (2019). Molecular dependencies of dynamic stiffening and strengthening through high strain rate microparticle impact of polyurethane and polyurea elastomers. Appl. Phys. Lett..

[46] Yao K., Chu D., Li T., Liu Z., Guo B.-H., Xu J., Zhuang Z. (2020). Atomic-scale simulation of hugoniot relations and energy dissipation of polyurea under high-speed shock. Eng. Comput..

[47] Yao K., Liu Z., Li T., Guo B., Zhuang Z. (2020). Mesoscale structure-based investigation of polyurea dynamic modulus and shock-wave dissipation. Polymer.

[48] Akl W., Nouh M., Aldraihem O., Baz A. (2019). Energy dissipation characteristics of polyurea and polyurea/carbon black composites. Mech. Time-Dependent Mater..

[49] Chen D., Wu H., Wei J.S., Xu S.L., Fang Q. (2022). Nonlinear visco-hyperelastic tensile constitutive model of spray polyurea within wide strain-rate range. Int. J. Impact Eng..

[50] Chen Y., Guo H., Sun M., Lv X. (2022). Tensile Mechanical Properties and Dynamic Constitutive Model of Polyurea Elastomer under Different Strain Rates. Polymers.

[51] Wang X., Ji H., Li X., Sun S., Zhang Q., Shim V., Lu T.J. (2022). Static and dynamic compressive and tensile response of highly stretchable polyurea. Int. J. Impact Eng..

[52] Wang H., Deng X., Wu H., Pi A., Li J., Huang F. (2019). Investigating the dynamic mechanical behaviors of polyurea through experimentation and modeling. Def. Technol..

[53] Fan J., Chen A. (2019). Studying a Flexible Polyurethane Elastomer with Improved Impact-Resistant Performance. Polymers.

[54] Wu G., Wang X., Ji C., Gao Z., Jiang T., Zhao C., Liu Y. (2021). Anti-blast properties of 6063-T5 aluminum alloy circular tubes coated with polyurea elastomer: Experiments and numerical simulations. Thin-Walled Struct..

[55] Li B., Zhang Z., Wang X., Liu X. (2019). Investigation on the Debonding Failure Model of Anchored Polyurea Coating under a High-Velocity Water Flow and Its Application. Sustainability.

[56] He L., Attard T.L., Zhou H., Brooks A. (2019). Integrating energy transferability into the connection-detail of coastal bridges using reinforced interfacial epoxy-polyurea reaction matrix composite. Compos. Struct..

[57] Rijensky O., Rittel D. (2021). Numerical investigation of polyurea coated aluminum plates under hydrodynamic shocks. Thin-Walled Struct..

[58] Dewapriya M., Miller R. (2020). Molecular dynamics study of the mechanical behaviour of ultrathin polymer–metal multilayers under extreme dynamic conditions. Comput. Mater. Sci..

[59] Chen C., Wang X., Hou H., Cheng Y., Zhang P., Liu J. (2020). Effect of strength matching on failure characteristics of polyurea coated thin metal plates under localized air blast loading: Experiment and numerical analysis. Thin-Walled Struct..

[60] Nantasetphong W., Jia Z., Hasan M., Amirkhizi A., Nemat-Nasser S. (2018). A New Technique for Characterization of Low Impedance Materials at Acoustic Frequencies. Exp. Mech..

[61] Yao K., Liu Z., Zhuang Z. (2022). Atomic insights into shock-induced spalling of polyurea by molecular dynamics simulation. Extrem. Mech. Lett..

[62] Rahimzadeh T., Arruda E.M., Thouless M.D. (2015). Design of armor for protection against blast and impact. J. Mech. Phys. Solids.

[63] Chu D., Li Z., Yao K., Wang Y., Tian R., Zhuang Z., Liu Z. (2022). Studying the strengthening mechanism and thickness effect of elastomer coating on the ballistic-resistance of the polyurea-coated steel plate. Int. J. Impact Eng..

[64] Wu G., Wang X., Ji C., Liu Q., Xie X., Zhao C., Liu P. (2022). Damage response of polyurea-coated steel plates under combined blast and fragments loading. J. Constr. Steel Res..

[65] Gu M., Ling X., Wang H., Yu A., Chen G. (2019). Experimental and Numerical Study of Polymer-Retrofitted Masonry Walls under Gas Explosions. Processes.

[66] Zhang L., Wang Y., Wang X., Ji C., Gu J., Zhao C. (2022). Investigation on the influence mechanism of polyurea material property on the blast resistance of polyurea-steel composite plate. Structures.

[67] Chen Y., Jian Z., Xiao S., Wang L., Li X., Wang K., Deng H., Hu W. (2021). Molecular dynamics simulation of shock wave propagation and spall failure in single crystal copper under cylindrical impact. Appl. Phys. Express.

[68] Dewapriya M., Miller R. (2021). Energy absorption mechanisms of nanoscopic multilayer structures under ballistic impact loading. Comput. Mater. Sci..

[69] Lee T.-H., Park J.-H., Yang D.-H., Kim J.-H.J., Noor N.B.M. (2022). Material enhancements of newly developed stiff type polyurea for retrofitting of concrete structures. Case Stud. Constr. Mater..

[70] Fan W., Xie R., Davidson M., Yin H., Lai K., Wu Q. (2023). Crashworthiness and energy absorption of UHPFRC-steel composite sandwich structures under impact loading. Compos. Struct..

[71] Wang X., Yue Z., Xu X., Zhao Z., Ji H., Zhu M., Wang P., Zhang Q., Lu T.J. (2023). Ballistic impact response of elastomer-retrofitted corrugated core sandwich panels. Int. J. Impact Eng..

[72] Wu J., Liu Z., Yu J., Xu S. (2022). Experimental and numerical investigation of normal reinforced concrete panel strengthened with polyurea under near-field explosion. J. Build. Eng..

[73] Fallon C., McShane G. (2019). Impact mitigating capabilities of a spray-on elastomer coating applied to concrete. Int. J. Impact Eng..

[74] El-Sisi A.E., Saucier A., Salim H.A., Hoemann J.M. (2019). Experimental and Numerical Evaluation of Reinforced Concrete Walls Retrofit Systems for Blast Mitigation. J. Perform. Constr. Facil..

[75] Iqbal N., Sharma P., Kumar D., Roy P. (2018). Protective polyurea coatings for enhanced blast survivability of concrete. Constr. Build. Mater..

[76] Shi C., Zhang J., Wang X., Liu F., Chen W., Ma Z., Liang H., Zhao Y. (2023). Improving the Impact Resistance and Antisplash of Civil Air Defense Wall: Experiments and Finite-Element Simulation. J. Struct. Eng..

[77] Lyu P., Fang Z., Wang X., Huang W., Zhang R., Sang Y., Sun P. (2022). Explosion Test and Numerical Simulation of Coated Reinforced Concrete Slab Based on BLAST Mitigation Polyurea Coating Performance. Materials.

[78] Chen Y.-S., Wang B., Zhang B., Zheng Q., Zhou J.-N., Jin F.-N., Fan H.-L. (2020). Polyurea coating for foamed concrete panel: An efficient way to resist explosion. Def. Technol..

[79] Zhang Y., Hu J., Zhao W., Hu F., Yu X. (2023). Numerical Simulation of the Blast Resistance of SPUA Retrofitted CMU Masonry Walls. Buildings.

[80] Ji L., Wang P., Cai Y., Shang W., Zu X. (2022). Blast Resistance of 240 mm Building Wall Coated with Polyurea Elastomer. Materials.

[81] Gu M., Wang H., Yu A., Wang H., Ling X., Chen G. (2022). Research on dynamic behavior and gas explosion resistance of polyurea. Mater. Today Commun..

[82] Liu Q., Guo B., Chen P., Zhai H., Guo Y., Tang S. (2022). Experimental investigation blast resistance of CFRP/polyurea composite plates under blast loading. Thin-Walled Struct..

[83] Li Z., Chen L., Fang Q., Chen W., Hao H., Zhu R., Zheng K. (2019). Experimental and numerical study on CFRP strip strengthened clay brick masonry walls subjected to vented gas explosions. Int. J. Impact Eng..

[84] Wu G., Ji C., Wang X., Gao F.-Y., Zhao C.-X., Liu Y.-J., Yang G.-L. (2021). Blast response of clay brick masonry unit walls unreinforced and reinforced with polyurea elastomer. Def. Technol..

[85] Zhu H., Wang X., Wang Y., Ji C., Wu G., Zhang L., Han Z. (2022). Damage behavior and assessment of polyurea sprayed reinforced clay brick masonry walls subjected to close-in blast loads. Int. J. Impact Eng..

[86] Lantz L., Maynez J., Cook W., Wilson C.M.D. (2016). Blast Protection of Unreinforced Masonry Walls: A State-of-the-Art Review. Adv. Civ. Eng..

[87] Akın E., Tunaboyu O., Avşar Ö. (2020). Axial behavior of FRP confined low-strength concrete with polyurea. Structures.

[88] Song J.-H., Lee E.-T., Eun H.-C. (2020). A Study on the Strengthening Performance of Concrete Beam by Fiber-Reinforced Polyurea (FRPU) Reinforcement. Adv. Civ. Eng..

[89] Szafran J., Matusiak A., Rzeszut K., Jankowiak I. (2023). The influence of a polyurea coating on bent reinforced concrete beams with various reinforcement ratios. Bull. Pol. Acad. Sci.-Tech. Sci..

[90] Song J.-H., Lee E.-T., Eun H.-C. (2019). A Study on the Improvement of Structural Performance by Glass Fiber-Reinforced Polyurea (GFRPU) Reinforcement. Adv. Civ. Eng..

[91] Shi S., Liao Y., Peng X., Liang C., Sun J. (2019). Behavior of polyurea-woven glass fiber mesh composite reinforced RC slabs under contact explosion. Int. J. Impact Eng..

[92] Liu Y., Wang P., Jin F., He H., Zhou Y., Chen H., Zhou J., Wang B., Fan H. (2021). Blast responses of polyurea-coated concrete arches. Arch. Civ. Mech. Eng..

[93] Yue Z., Zhou J., Kong X., Xu Y., Chen Y., Wang B., Huang Y., Wang P. (2023). Anti-Blast Performance of Polyurea-Coated Concrete Arch Structures. Polymers.

[94] Liu Q., Guo B.Q., Chen P.W., Su J.J., Arab A., Ding G., Yan G.H., Jiang H.Y., Guo F. (2021). length Investigating ballistic resistance of CFRP/polyurea composite plates subjected to ballistic impact. Thin-Walled Struct..

[95] Petre R., Zecheru T., Ginghina R. (2021). Dynamic Tests on Polyurea-Based Hybrid Composites for Ballistic Protection. Mater. Plast..

[96] Bucur F., Trana E., Rotariu A. (2019). Numerical and Experimental Study on the Locally Blast Loaded Polyurea Coated Steel Plates. Mater. Plast..

[97] Li Y., Chen C., Hou H., Cheng Y., Gao H., Zhang P., Liu T. (2019). The Influence of Spraying Strategy on the Dynamic Response of Polyurea-Coated Metal Plates to Localized Air Blast Loading: Experimental Investigations. Polymers.

[98] Hou H., Chen C., Cheng Y., Zhang P., Tian X., Liu T., Wang J. (2019). Effect of structural configuration on air blast resistance of polyurea-coated composite steel plates: Experimental studies. Mater. Des..

[99] Mohotti D., Fernando P., Weerasinghe D., Remennikov A. (2020). Evaluation of effectiveness of polymer coatings in reducing blast-induced deformation of steel plates. Def. Technol..

[100] Stergiou T., Baxevanakis K.P., Roy A., Sazhenkov N.A., Nikhamkin M.S., Silberschmidt V.V. (2021). Impact of polyurea-coated metallic targets: Computational framework. Compos. Struct..

[101] Zhang P., Wang Z., Zhao P., Zhang L., Jin X., Xu Y. (2019). Experimental investigation on ballistic resistance of polyurea coated steel plates subjected to fragment impact. Thin-Walled Struct..

[102] Toader G., Diacon A., Rusen E., Rizea F., Teodorescu M., Stanescu P.O., Damian C., Rotariu A., Trana E., Bucur F. (2021). A Facile Synthesis Route of Hybrid Polyurea-Polyurethane-MWCNTs Nanocomposite Coatings for Ballistic Protection and Experimental Testing in Dynamic Regime. Polymers.

[103] Liu Q.-Q., Wang S.-P., Lin X., Cui P., Zhang S. (2020). Numerical simulation on the anti-penetration performance of polyurea-core Weldox 460 E steel sandwich plates. Compos. Struct..

[104] Youssef G., Reed N., Huynh N.U., Rosenow B., Manlulu K. (2020). Experimentally-validated predictions of impact response of polyurea foams using viscoelasticity based on bulk properties. Mech. Mater..

[105] Reed N., Huynh N.U., Rosenow B., Manlulu K., Youssef G. (2019). Synthesis and characterization of elastomeric polyurea foam. J. Appl. Polym. Sci..

[106] Youssef G., Kokash Y., Uddin K.Z., Koohbor B. (2022). Density-Dependent Impact Resilience and Auxeticity of Elastomeric Polyurea Foams. Adv. Eng. Mater..

[107] Ramirez B.J., Gupta V. (2019). High tear strength polyurea foams with low compression set and shrinkage properties at elevated temperatures. Int. J. Mech. Sci..

[108] Do S., Huynh N.U., Reed N., Shaik A.M., Nacy S., Youssef G. (2020). Partially-Perforated Self-Reinforced Polyurea Foams. Appl. Sci..

[109] He Q., Cao S., Wang Y., Xuan S., Wang P., Gong X. (2018). Impact resistance of shear thickening fluid/Kevlar composite treated with shear-stiffening gel. Compos. Part A Appl. Sci. Manuf..

[110] Haris A., Lee H.P., Tan V.B.C. (2018). An experimental study on shock wave mitigation capability of polyurea and shear thickening fluid based suspension pads. Def. Technol..

[111] Jafari S., Nia A.A. (2022). An improvement on the Florence analytical equation for predicting the ballistic limit velocity of ceramic–aluminum targets and its development to ceramic–aluminum–polyurea panels. J. Braz. Soc. Mech. Sci. Eng..

[112] Jafari S., Nia A.A. (2023). Numerical and analytical investigation of ballistic performance of composite targets with ceramic-polyurea-metal layers and optimization of the layer thicknesses. J. Aust. Ceram. Soc..

[113] Si P., Liu Y., Yan J., Bai F., Shi Z., Huang F. (2023). Effect of polyurea layer on ballistic behavior of ceramic/metal armor. Structures.

[114] Wang Y., Jia X., Huang Z., Yang C., Xudong Z. (2022). Polyurea-coated ceramic-aluminum composite plates subjected to low velocity large fragment impact. Mater. Today Commun..

[115] Zou G., Wu S., Yan A., Chang Z., Li Y., Zhang Z. (2023). Penetration resistance of ceramic/PUE/GFRP multi-layered composite structure. Compos. Struct..

[116] Xiao Y., Tang Z., Hong X. (2021). Low velocity impact resistance of ceramic/polyurea composite plates: Experimental study. J. Mech. Sci. Technol..

[117] Zhang G., Liu Y., Lv Z., Wang J., Zhang W., Wu Y. (2021). Research on impact resistance of ceramic matrix composites. Compos. Struct..

[118] Zhang X., Meng Q., Zhang K., Zhu R., Qu Z., Li Y., He R. (2023). 3D-printed bioinspired Al_2_O_3_/polyurea dual-phase architecture with high robustness, energy absorption, and cyclic life. Chem. Eng. J..

[119] Dewapriya M., Miller R. (2021). Molecular dynamics study of the penetration resistance of multilayer polymer/ceramic nanocomposites under supersonic projectile impacts. Extrem. Mech. Lett..

[120] Yang F., Li Z., Liu Z., Zhuang Z. (2021). Shock Loading Mitigation Performance and Mechanism of the PE/Wood/PU/Foam Structures. Int. J. Impact Eng..

[121] Grujicic M., He T., Pandurangan B., Svingala F.R., Settles G.S., Hargather M.J. (2012). Experimental Characterization and Material-Model Development for Microphase-Segregated Polyurea: An Overview. J. Mater. Eng. Perform..

[122] Dai L.-H., Wu C., An F.-J., Liao S.-S. (2018). Experimental Investigation of Polyurea-Coated Steel Plates at Underwater Explosive Loading. Adv. Mater. Sci. Eng..

[123] Rijensky O., Rittel D. (2020). Experimental investigation of polyurea coated aluminum plates under strong hydrodynamic shocks. Thin-Walled Struct..

[124] Bijanzad A., Abdulwahab M., Lazoglu I., Ensarioglu C., Cakir M.C. (2022). Effect of polyurea coating on the ductility of aluminum foam. Mater. Today Commun..

[125] Liu X., Sun Q., Sun Y., Xie Y. (2023). Investigation on energy-absorption-based protection mechanism of polyurea-coated aluminum alloy composite (PCAA) plate under impact loading by K9 glass projectile. Mech. Mater..

[126] Ren J., Zhou Y., Zhao Z. (2023). Response of elastomer-coated aluminum plate under foam projectile impact. Compos. Struct..

[127] Fang C., Linzell D.G., Yosef T.Y., Rasmussen J.D. (2022). Numerical Modeling and Performance Assessment of Bridge Column Strengthened by FRP and Polyurea under Combined Collision and Blast Loading. J. Compos. Constr..

[128] Fang C., Yosef T.Y., Linzell D.G. (2023). Multi-Hazard-Resistant Behavior of CFRP- and Polyurea-Retrofitted Reinforced Concrete Two-Column Piers under Combined Collision-Blast Loading. Materials.

[129] Kosiński S., Gonsior M., Krzyżanowski P., Rykowska I. (2021). New Hybrid Polyurea-Polyurethane Elastomers with Antistatic Properties and an Influence of Various Additives on Their Physicochemical Properties. Molecules.

[130] Kiran K.K., Ahmad S., Al-Osta M.A., Bahraq A.A. (2022). Performance of the polyurea-coated steel tank under air blast load: A numerical study. Arch. Civ. Mech. Eng..

[131] Jiang Y., Zhang B., Wei J., Wang W. (2020). Study on the dynamic response of polyurea coated steel tank subjected to blast loadings. J. Loss Prev. Process. Ind..

[132] Jiang Y., Zhang B., Wang L., Wei J., Wang W. (2021). Dynamic response of polyurea coated thin steel storage tank to long duration blast loadings. Thin-Walled Struct..

[133] Tao C., Ji C., Zhao C., Wang X., Wang Y. (2023). Mechanism of polyurea in protecting liquid-filled square aluminum tube from the impact of high-velocity projectile. Thin-Walled Struct..

[134] Wu G., Wang X., Ji C., Liu Q., Gao Z., Zhang K., Zhao C. (2021). Experimental and numerical simulation study on polyurea-coated fuel tank subjected to combined action of blast shock waves and fragments. Thin-Walled Struct..

[135] Wang X., Ji C., Wu G., Wang Y., Zhu H. (2022). Damage response of high elastic polyurea coated liquid-filled tank subjected to close-in blast induced by charge with prefabricated fragments. Int. J. Impact Eng..

[136] Mostofi T.M., Sayah-Badkhor M., Rezasefat M., Ozbakkaloglu T., Babaei H. (2020). Gas mixture detonation load on polyurea-coated aluminum plates. Thin-Walled Struct..

[137] Peng Q., Sun X., Liu Z., Jin J., Yu H., Yin Y. (2023). Sprayed-Polyurea-Modified Asphalt: Optimal Preparation Parameters, Rheological Properties and Thermal Properties. Coatings.

[138] Sun X., Yuan Z., Huang Z., Xu Q., Zhu Y., Xu X., Yuan J., Liu Z., Zhang Y., Chen Q. (2023). Applying solution of spray polyurea elastomer in asphalt binder: Feasibility analysis and DSR study based on the MSCR and LAS tests. Nanotechnol. Rev..

[139] Özalp F., Yilmaz H.D., Zeytun S., Akcay B. (2023). Effects of EAF Slag, Steel Fiber, and Polyurea Coating on Mechanical Properties and Sulfuric Acid Resistance of Concrete Pipes. J. Pipeline Syst. Eng. Pr..

[140] Gauch E., LeBlanc J., Shukla A. (2018). Near field underwater explosion response of polyurea coated composite cylinders. Compos. Struct..

[141] Liu J., An F., Niu Z., Zhang L., Feng B., Li Y., Wu C. (2022). Study on the blast-resistance of polyurea-steel plates subjected to underwater explosion. Ocean Eng..

[142] Duda M., Pach J., Lesiuk G. (2019). Influence of Polyurea Composite Coating on Selected Mechanical Properties of AISI 304 Steel. Materials.

[143] Jiang Y., Zhang B., Wei J., Wang W. (2019). Study on the impact resistance of polyurea-steel composite plates to low velocity impact. Int. J. Impact Eng..

